# Pou3f1 mediates the effect of Nfatc3 on ulcerative colitis-associated colorectal cancer by regulating inflammation

**DOI:** 10.1186/s11658-022-00374-0

**Published:** 2022-09-05

**Authors:** Yan Lin, Dongxu Wang, Hong Zhao, Dongyue Li, Xinning Li, Lianjie Lin

**Affiliations:** 1 Department of Gastroenterology, Shengjing Hospital of China Medical University, 36 Sanhao Street, Heping District, 110004 Shenyang, China; 2 Department of Gastroenterology, The Second Affiliated Hospital of Shenyang Medical College, Shenyang, China; 3 Department of Respiratory, Ansteel Group General Hospital, Anshan, China; 4 Medical Oncology Ward, Tieling Central Hospital, Tieling, China

**Keywords:** Ulcerative colitis-associated colorectal cancer, Macrophage, Pou3f1, Nfatc3, Inflammation

## Abstract

**Background:**

Ulcerative colitis-associated colorectal cancer (UC-CRC) is an important complication of ulcerative colitis. Pou3f1 (POU class 3 homeobox 1) is a critical regulator for developmental events and cellular biological processes. However, the role of Pou3f1 in the development of UC-CRC is unclear.

**Methods:**

In vivo, a UC-CRC mouse model was induced by azoxymethane (AOM) and dextran sulfate sodium (DSS). Body weight, colon length, mucosal damage, tumor formation, and survival rate were assessed to determine the progression of UC-CRC. Western blot, quantitative real-time PCR, ELISA, immunohistochemistry, immunofluorescence and TUNEL were performed to examine the severity of inflammation and tumorigenesis. In vitro, LPS-treated mouse bone marrow-derived macrophages (BMDMs) and RAW264.7 cells were used to study the role of Pou3f1 in inflammation. ChIP and luciferase reporter assays were used to confirm the interaction between Nfatc3 and Pou3f1.

**Results:**

Pou3f1 expression was increased in the colons of UC-CRC mice, and its inhibition attenuated mucosal injury, reduced colon tumorigenesis and increased survival ratio. Knockdown of Pou3f1 suppressed cell proliferation and increased cell death in colon tumors. Both the in vivo and in vitro results showed that Pou3f1 depletion reduced the production of proinflammation mediators. In addition, ChIP and luciferase reporter assays demonstrated that Nfatc3 directly bound with the Pou3f1 promoter to induce its expression. The effect of Nfatc3 on the inflammatory response in macrophages was suppressed by Pou3f1 knockdown.

**Conclusion:**

Overall, it outlines that Pou3f1 mediates the role of Nfatc3 in regulating macrophage inflammation and carcinogenesis in UC-CRC development.

**Supplementary Information:**

The online version contains supplementary material available at 10.1186/s11658-022-00374-0.

## Background

Colorectal cancer (CRC) is one of the most prevalent cancers with high mortality worldwide, and its pathogenesis is not fully understood [1]. The epidemiological study suggests that chronic inflammation is an essential driving factor for CRC development [2]. Patients with ulcerative colitis may be more likely to develop ulcerative colitis-associated colorectal cancer (UC-CRC) than healthy individuals [3–5].

Chronic inflammation that induces intestinal mucosa injury and carcinogenesis is a critical contributor to UC-CRC [6, 7]. It is well-demonstrated that macrophages are the main inflammatory cells that involve in tumorigenesis [8, 9]. The activated macrophages produce several inflammatory mediators, such as interleukin-6 (IL-6), interleukin-1β (IL-1β), tumor necrosis factor-α (TNF-α) and monocyte chemotactic protein-1 (MCP-1), and disturb the tumor microenvironment [10]. It is recognized that the reactive oxygen accumulation caused by persistent inflammation participates in regulating cell proliferation and tumor progression [11, 12]. Therefore, targeting macrophages might be a potential strategy for UC-CRC prevention or therapy.

Nfatc3 (also known as Nfat4 or Nfatx) is a member of the nuclear factor of activated T cells (Nfat) family, and it has been suggested to be relevant to inflammation and the progression of cancer [13, 14]. Awla et al. reported that Nfatc3 inhibition reduced myeloperoxidase (MPO) activity and inflammatory gene expression in acute pancreatitis [15]. Nfatc3 was highly expressed in the hypothalamus of high-fat diet-treated mice and induced a significant upregulation of inflammatory regulators [16]. Our previous study demonstrated that Nfatc3 promoted the inflammatory response and tumorigenesis in azoxymethane/dextran sulfate sodium (AOM/DSS)-induced mice [17]. However, the underlying mechanism by which Nfatc3 induces UC-CRC progression is unknown.

Pou3f1 (also named as Oct-6, SCIP or Tst-1) is a member of the Pit-Oct-Unc (POU) family, and it regulates embryogenesis, epidermal differentiation and neurogenesis [18–21]. Fionda et al. found that inhibition of Pou3f1 combined with doxorubicin induced G2-cell cycle arrest and cell apoptosis in non-small-cell lung carcinoma [22]. Hofmann et al. demonstrated that Pou3f1 expression was increased in fibroblasts and macrophages in the presence of IFN-β and IFN-γ [23], implying its potential in regulating immune responses. However, the role of Pou3f1 in the development of UC-CRC remains unclear. Thus, the present work aims to clarify the importance of Pou3f1 in UC-CRC and the involvement of Nfatc3.

## Methods

### Animal models

**Fig. 1:**
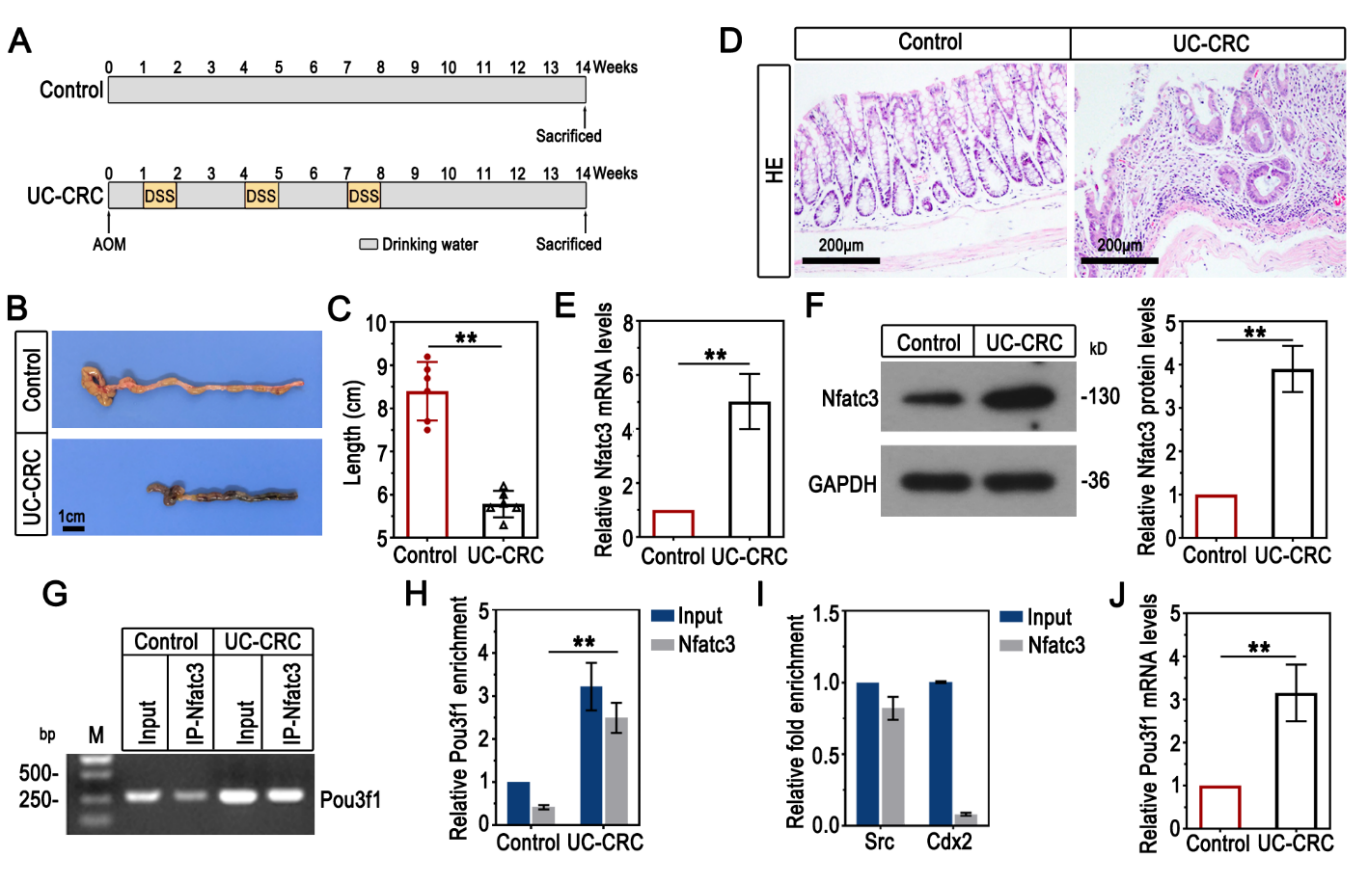
Pou3f1 physically interacted with Nfatc3, and it was upregulated in colons of UC-CRC mice. (A) The schematic diagram for animal model protocol. (B-C) The colon length in mice was recorded. (D) Representative images of HE-stained colon sections. (E) Nfatc3 mRNA levels in colons were determined using qPCR. (F) Western blot analysis for Nfatc3 protein levels in colons and quantitative results. (G-H) ChIP-PCR and ChIP-qPCR were performed to determine the interaction between Nfatc3 and Pou3f1 in colons. M: Marker. (I) ChIP-qPCR assay showed that Nfatc3 bound to the Src promoter, rather than the Cdx2 promoter in colon tissues. Src was used as a positive control. Cdx2 was used as a negative control. (J) The relative mRNA levels of Pou3f1 were detected by qPCR analysis. Data were from n = 6 mice per group. Values were mean ± SD. **, p < 0.01

**Fig. 2:**
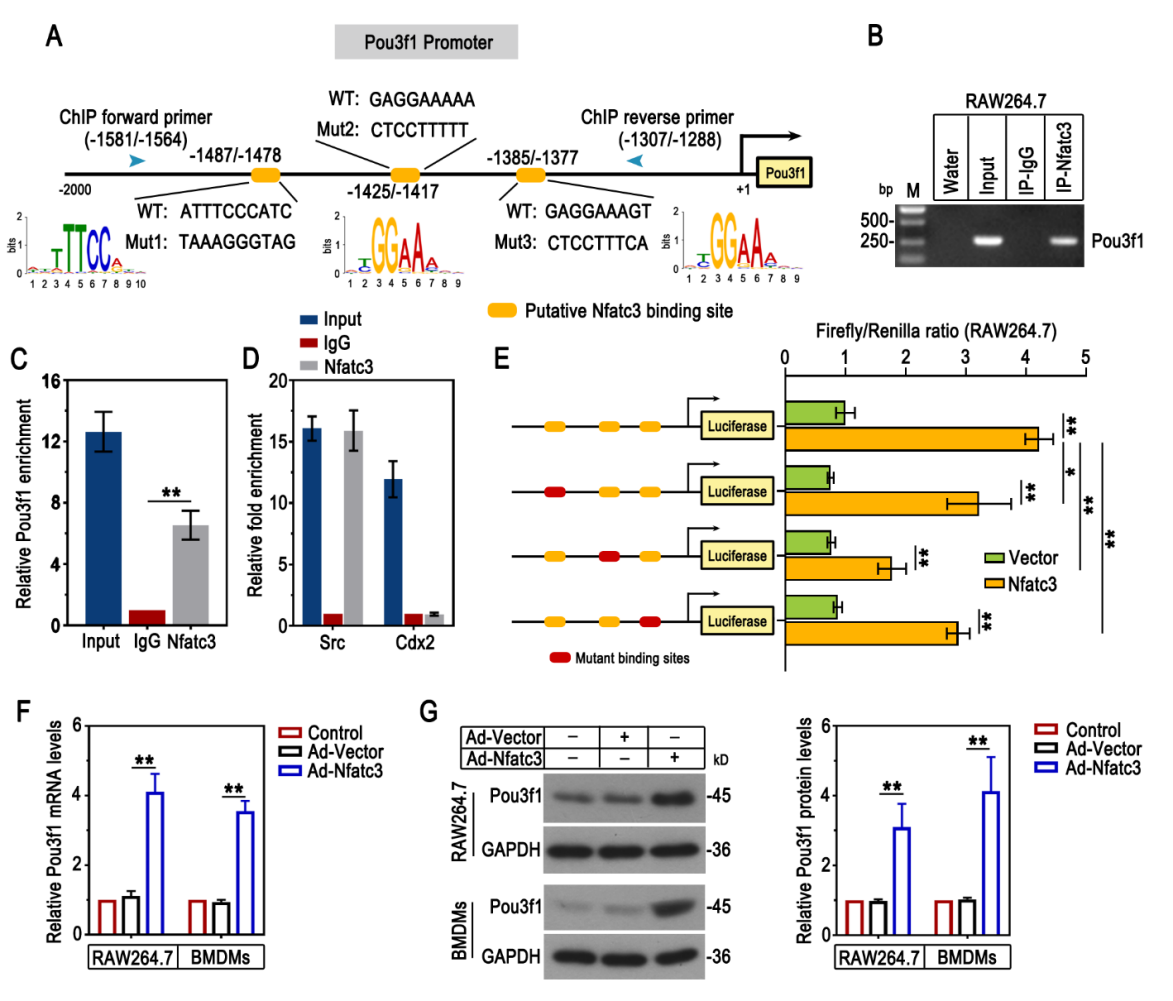
Pou3f1 was identified as a direct transcriptional target of Nfatc3. (A) The schematic of the potential motifs and binding sites of Nfatc3 in the Pou3f1 promoter region. (B-C) ChIP-PCR and ChIP-qPCR were performed to demonstrate the direct interaction between Nfatc3 and Pou3f1 in RAW264.7 cells. M: Marker. (D) ChIP-qPCR assay demonstrated that Nfatc3 bound to the Src promoter, rather than the Cdx2 promoter in RAW264.7 cells. Src was used as a positive control. Cdx2 was used as a negative control. (E) The relative luciferase activity was measured by luciferase reporter assay in RAW264.7 cells. (F) The mRNA levels of Pou3f1 in RAW264.7 cells and BMDMs were measured by qPCR. (G) Western blot analysis and quantification results for Pou3f1 protein levels in RAW264.7 cells and BMDMs. n = 3. Values were mean ± SD. *, p < 0.05; ** p < 0.01

Six- to eight-week-old male C57BL/6 mice (15–20 g) were maintained in a standard environment at 22 ± 1 °C on a 12-h light/dark cycle. After adaptive feeding for 1 week, mice were subjected to the modeling. The experimental protocol for UC-CRC model establishment was illustrated in Fig. 1A. In brief, mice were injected with 10 mg/kg AOM (A5486, Sigma, China) intraperitoneally. One week later, mice were given 3% DSS (MP Biomedicals, China) in drinking water for consecutive 7 days, followed by normal drinking water for consecutive 14 days. The DSS-normal drinking water cycle was repeated for 3 times. To determine the role of Pou3f1 in UC-CRC development, the adeno-associated virus 9 vectors carrying short hairpin RNA targeting Pou3f1 (AAV-shPou3f1) and negative control (AAV-shNC) were constructed. The AAV particles were administrated to UC-CRC mice by coloclysis 2 weeks prior to AOM treatment (Fig. 2A).

Several indicators for animal health were monitored weekly, including body weight, drinking water/food consumption, stool, diarrhea, and rectal bleeding. The disease activity index was determined as previously described [24].

### Cell treatment

RAW264.7 cells (ZQ0098) were purchased from ZhongQiaoXinZhou (China) and cultured in Dulbecco’s modified eagle medium (DMEM; G4510, Servicebio, China) containing 10% FBS with 5% CO_2_ at 37 °C. Mouse bone marrow-derived macrophages (BMDMs; iCell-0060a, iCell Bioscience Inc, China) were incubated in a special complete medium (CM-M141, Procell, China) with 5% CO_2_ at 37 °C.

For gene overexpression or knockdown in vitro, the adenovirus vectors expressing Nfatc3/control (Ad-Nfatc3/Ad-Vector) and short hairpin RNA targeting Pou3f1/control (Ad-shPou3f1/Ad-shNC) were prepared to infect RAW264.7 cells or BMDMs. The infection was conducted for 48 h via Lipofectamine 3000 (L3000-008, Invitrogen, USA). After the 48-h infection, RAW264.7 cells were treated with 100 ng/ml lipopolysaccharide (LPS; L8880, Solarbio, China) for 3 h to induce an acute inflammatory response.

### Chromatin immunoprecipitation (ChIP) assay

ChIP assay was performed using the Tissue ChIP Kit (WLA122, Wanleibio, China) or the Cell ChIP Kit (WLA106a, Wanleibio) following the manufacturers’ protocols. Colon tissues were cut into 1–3 mm pieces, crosslinked with 1% formaldehyde and placed in a glass homogenizer for homogenizing. RAW264.7 cells were crosslinked with 1% formaldehyde. Chromatin was immunoprecipitated using Nfatc3 antibody (700 µg/mL; 18222-1-AP, Proteintech, China) or normal rabbit IgG antibody (0.5 µg/µL). The precipitated DNAs were subjected to PCR or qPCR reaction. The primer sequences (5’-3’) targeting Pou3f1 were: forward, AGCCAAATGATGGACAGA; reverse, GGTATGAGATAGAGGGAGTG.

### Luciferase reporter assay

To assess the effect of Nfatc3 on Pou3f1 transcriptional activity, the fragments of the wild type or mutant Pou3f1 promoter region were inserted into pGL3-Basic luciferase reporter vectors. The Pou3f1 luciferase reporters were co-transfected with Nfatc3 overexpression plasmids (Nfatc3-OE) into RAW264.7 cells using Lipofectamine 3000. Forty-eight hours later, firefly and renilla luciferase activities were measured using the Luciferase Reporter Gene Assay Kit (KGAF040, KeyGEN BioTECH, China).

### Measurement of ROS levels

The ROS Assay Kit (S0033, Beyotime, China) was utilized to measure intracellular ROS contents in vitro. Cells were washed in PBS twice, and incubated with DCFH-DA solution at 37 °C. After resuspending in PBS, the fluorescence intensity was determined by flow cytometry (NovoCyte, AceaBio, USA).

To detect ROS production in vivo, colon tissues were cut into small pieces and crushed with a homogenizer in PBS to prepare single-cell suspensions. After incubation with DCFH-DA solution (E004, JianCheng, China) at 37 °C, the single-cell suspensions were examined with a multimode Reader (Synergy H1, Biotek, USA). The fluorescent intensity of ROS was measured at the excitation wavelength of 490 nm and the emission wavelength of 540 nm.

### Enzyme-linked immunosorbent assay (ELISA)

For ELISA analysis, colon segments were mechanically crushed in saline and homogenate supernatants were harvested after centrifugation. The protein concentration of colon supernatants was quantified with a BCA Protein Assay Kit (PC0020, Solarbio). The amount of IL-1β (EK201B, Multi Sciences, China), monocyte chemotactic protein-1 (MCP-1; EK287, Multi Sciences), and prostaglandin E_2_ (PGE_2_; EK8103, Multi Sciences) in colon homogenate was determined by ELISA.

### Determination of MPO activity

Colons were homogenized in the MPO buffer and the homogenate suspensions were extracted. The MPO activity was determined by an MPO Activity Assay Kit (A044, JianCheng).

### Histological staining

Colons were harvested, fixed in paraformaldehyde and embedded in paraffin. After deparaffinization, the 5-µm sections were stained with hematoxylin (H8070, Solarbio) and eosin (A600190, Sangon, China) (HE) for histological analysis as previously described [25]. For immunohistological staining, the sections were incubated with COX-2 antibody (1:50 dilution; A1253, ABclonal, China), and then incubated with HRP-conjugated Goat anti-Rabbit secondary antibody (1:500 dilution; 31,460, ThermoFisher Scientific, USA). For immunofluorescent staining, primary antibodies against Proliferating Cell Nuclear Antigen (PCNA; 1:50 dilution; A0264, ABclonal) and F4/80 (1:50 dilution; sc-377,009, Santa Cruz, USA) were applied. Sections were then incubated with secondary antibodies, including FITC-labeled Goat anti-Rabbit IgG antibody (1:200 dilution; A0562, Beyotime) and Cy3-labeled Goat anti-Mouse IgG antibody (1:50 dilution; A0521, Beyotime). TUNEL staining was performed to determine cell death in colons using the In Situ Cell Death Detection Kit (11,684,795,910, Roche, Switzerland) following the manufacturer’s instructions. Cell nuclei were counterstained using DAPI (D106471, Aladdin, China). Images were taken using the Olympus microscope (BX53, Olympus, Japan). The number of positive cells within 3 random ×400 fields/each Sect. (2 sections per mouse) was assessed by manual counting.

### Quantitative real-time PCR (qPCR)

Total RNA from the ground colon tissues in nitrogen and cultured cells was extracted using the TRIpure Reagent lysis buffer (RP1001, BioTeke, China), followed by cDNAs reverse transcription with the BeyoRT II M-MLV reverse transcriptase (D7160L, Beyotime). qPCR was performed with the SYBR Green reagent (SY1020, Solarbio) on the Exicycler 96 system (Bioneer, Korea). The primer sequences (5’-3’) were shown: Nfatc3 forward GGTAAAGAGCAGCACATA, Nfatc3 reverse TTGACTAGAGGCAGGATT; MCP-1 forward GCCTGCTGTTCACAGTTGCC, MCP-1 reverse CTGGACCCATTCCTTCTTGG; TNF-α forward CAGGCGGTGCCTATGTCTCA, TNF-α reverse GCTCCTCCACTTGGTGGTTT; IL-6 forward ATGGCAATTCTGATTGTATG, IL-6 reverse GACTCTGGCTTTGTCTTTCT; IL-1β forward CTCAACTGTGAAATGCCACC, IL-1β reverse GAGTGATACTGCCTGCCTGA; Pou3f1 forward CGTGTTCTCGCAGACCACCATC, Pou3f1 reverse CGCACCACCTCCTTCTCCAGTT; GAPDH forward TGTTCCTACCCCCAATGTGTCCGTC, GAPDH reverse CTGGTCCTCAGTGTAGCCCAAGATG. The relative gene expression was calculated with the 2^−*ΔΔ*Ct^ method and normalized to GAPDH.

### Western blot

Western blot analysis was performed as previously reported [26]. Total protein was prepared with the RIPA lysis reagent (R0010, Solarbio). After quantification with the BCA Protein Assay Kit, protein samples were separated on the SDS-PAGE gel and transferred onto PVDF membranes (IPVH00010, Millipore, USA). Membranes were incubated with primary antibodies, including Nfatc3 antibody (1:1000 dilution; 18222-1-AP, Proteintech), Pou3f1 antibody (1:1000 dilution; A19330, ABclonal), iNOS antibody (1:1000 dilution; A0312, Abclonal), COX-2 antibody (1:1000 dilution; A1253, Abclonal) and GAPDH antibody (1:10000 dilution; 60004-1-Ig, Proteintech). The Goat anti-Rabbit IgG/HRP antibody (1:3000 dilution; SE134, Solarbio) and Goat anti-Mouse IgG/HRP antibody (1:3000 dilution; SE131, Solarbio) were used as secondary antibodies. The protein signals were visualized using the ECL Western Blot Substrate (PE0010, Solarbio).

### Statistical analysis

All data were expressed as mean ± SD and analyzed using GraphPad Prism Software. Unpaired t test or one-way ANOVA followed by Bonferroni’s multiple comparisons test was used to determine the statistical difference. Survival analysis was performed using Kaplan-Meier with the Logrank test. p < 0.05 was identified as a significant difference.

## Results

**Pou3f1 was a direct target of Nfatc3, and it was upregulated in colons of UC-CRC mice**.

The UC-CRC animal model was established with AOM/DSS administration as Fig. 1A described. The shorten colon and histological epithelial damage were observed in mice treated with AOM/DSS (Fig. 1B-D). Consistent with our previous work [17], the increased mRNA and protein levels of Nfatc3 were found in colons of UC-CRC mice (Fig. 1E-F).

To illuminate the potential mechanism of Nfatc3 in UC-CRC, we demonstrated Pou3f1 as a direct target of Nfatc3. The results in Fig. 1G-H showed that Pou3f1 was enriched in UC-CRC, and Nfatc3 physically interacted with the Pou3f1 promoter. The positive control (known target gene Src) and negative control (known non-target gene Cdx2) were used to verify the interaction between Nfatc3 and Pou3f1 (Fig. 1I). In addition, we found that AOM/DSS treatment upregulated the mRNA levels of Pou3f1 (Fig. 1J).

The interaction of Nfatc3 and Pou3f1 was further investigated in vitro. The potential Nfatc3 consensus binding sites in Pou3f1 promoter sequence were represented in Fig. 2A. ChIP assays in vitro confirmed the direct interaction between Nfatc3 and Pou3f1 (Fig. 2B-D). As shown in Fig. 2E, Nfatc3 overexpression increased the luciferase activity of Pou3f1 when cells were transfected with the reporter plasmid containing wild-type Pou3f1 promoter sequence. In addition, Pou3f1 transcription activity was reduced in the presence of the mutant binding site (mutation 1, mutation 2 or mutation 3) in its promoter sequence. It evidenced the three binding sites of Nfatc3 in the Pou3f1 promoter by which Nfatc3 regulated Pou3f1 transcription. Adenovirus-mediated Nfatc3 overexpression was confirmed at both mRNA and protein levels in vitro (Supplementary Fig. 1A-B). Overexpression of Nfact3 increased the mRNA and protein expression of Pou3f1 (Fig. 2F-G). Thus, the results indicated that Nfatc3 directly bound to the Pou3f1 promoter to induce its expression.

### Pou3f1 knockdown ameliorated LPS-induced inflammation in macrophages

**Fig. 3:**
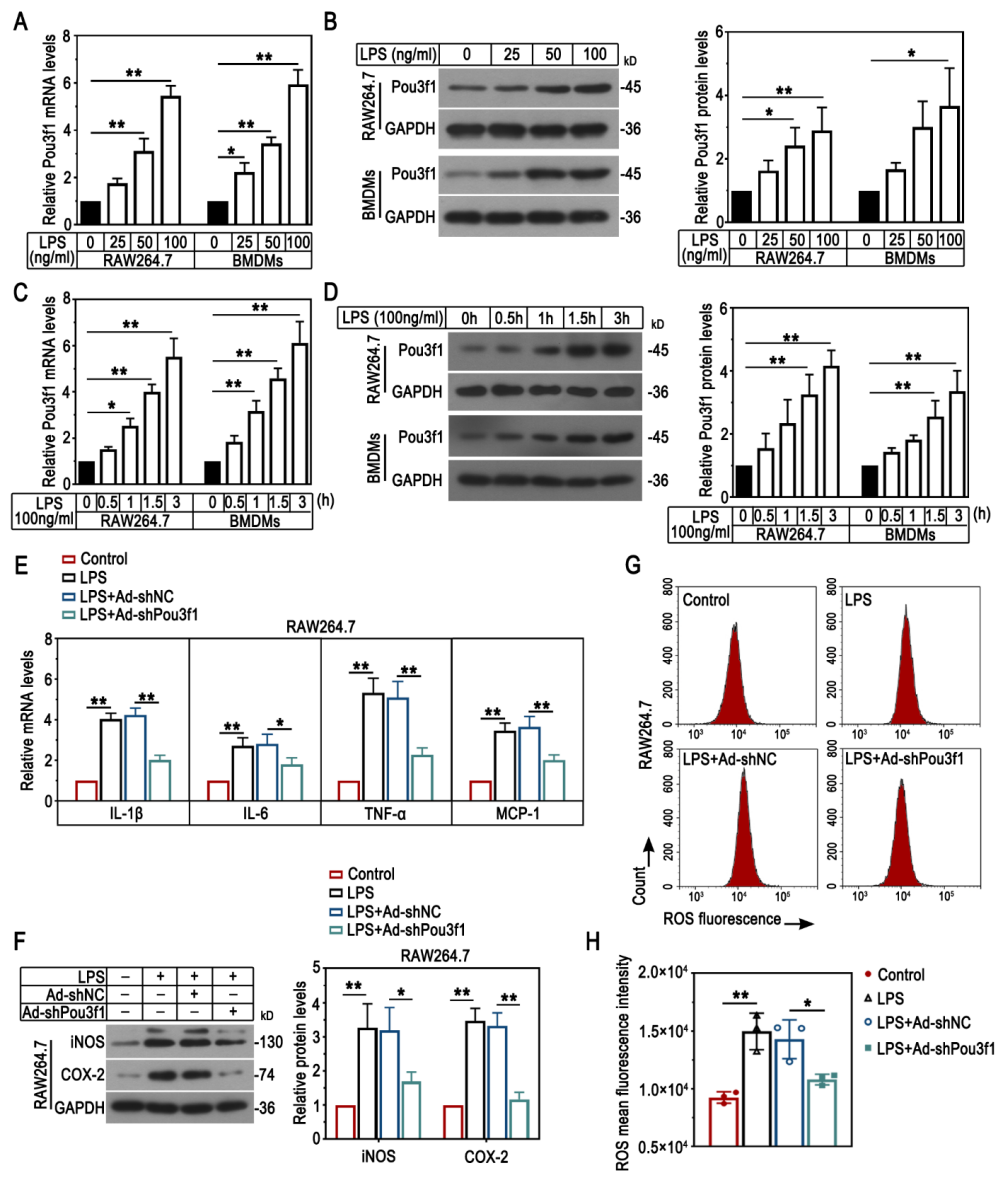
Pou3f1 knockdown ameliorated LPS-induced inflammation in macrophages. (A-D) The mRNA and protein levels of Pou3f1 in RAW264.7 cells and BMDMs in response to LPS were determined by qPCR and Western blot. (E) The relative mRNA levels of IL-1β, IL-6, TNF-α and MCP-1 were determined by qPCR. (F) The protein levels of iNOS and COX-2 were detected using Western blot. (G-H) Flow cytometry analysis and quantification results for the fluorescence intensity of ROS. n = 3. Values were mean ± SD. *, p < 0.05; **, p < 0.01

Macrophages in response to LPS were employed to mimic the inflammation of UC-CRC in vitro. Results showed that LPS treatment upregulated Pou3f1 mRNA and protein levels in both RAW264.7 cells and BMDMs in dose- and time-dependent manners (Fig. 3A-D). Nfatc3 protein expression displayed a similar trend in vitro (Supplementary Fig. 2A-B).

To investigate the effect of Pou3f1 on LPS-induced inflammation in macrophages, five pairs of siRNAs targeting Pou3f1 were transiently transfected into cells and the interference efficiency was confirmed (Supplementary Fig. 3A). Results showed that siPou3f1-1 displayed the most inhibitory effect on its mRNA expression. Thus, the adenovirus carrying the interference sequence of Pou3f1 (Ad-shPou3f1) was used to infect RAW264.7 cells, and the infection efficiency was confirmed (Supplementary Fig. 3B). As shown in Fig. 3E-F, the mRNA level of proinflammatory cytokines (IL-1β, IL-6, TNF-α and MCP-1) and the protein level of inflammatory mediators (iNOS and COX-2) were reduced by Pou3f1 inhibition in LPS-treated cells. Knockdown of Pou3f1 suppressed LPS-induced accumulation of ROS in vitro (Fig. 3G-H). Thus, the in vitro results showed that Pou3f1 was likely to aggravate the inflammatory response in macrophages.

### Pou3f1 was required for Nfatc3-induced inflammation in LPS-treated macrophages

**Fig. 4:**
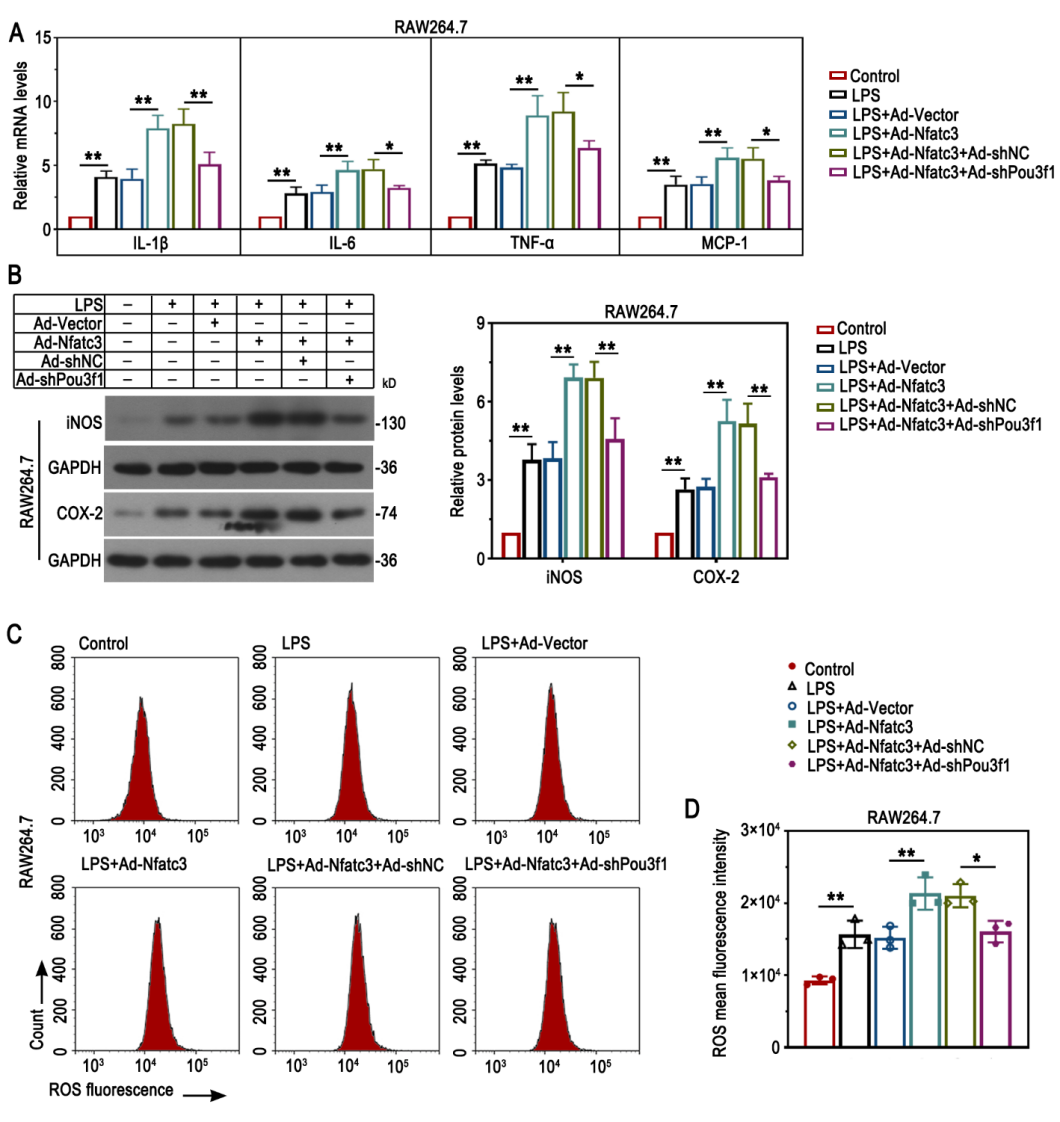
Pou3f1 was required for Nfatc3-induced inflammation in LPS-treated macrophages. (A) The relative mRNA levels of IL-1β, IL-6, TNF-α and MCP-1 were determined by qPCR. (B) The protein levels of iNOS and COX-2 were detected using Western blot. (C-D) Flow cytometry analysis and quantification results for the fluorescence intensity of ROS. n = 3. Values were mean ± SD. *, p < 0.05; **, p < 0.01

We next examined whether Pou3f1 is essential for Nfatc3-mediated inflammation in vitro. Results from Fig. 4A showed that Nfatc3 overexpression elevated the mRNA levels of proinflammatory cytokines in response to LPS, and knockdown of Pou3f1 attenuated this. Similar alterations in the protein expression of iNOS and COX-2 were demonstrated (Fig. 4B). Flow cytometry analysis suggested that Pou3f1 depletion ameliorated Nfatc3-induced ROS accumulation in LPS-treated macrophages (Fig. 4C-D). The results demonstrated that Pou3f1 was required for Nfatc3-induced inflammation in macrophages.

### Pou3f1 knockdown protected against mucosal injury and tumorigenesis of UC-CRC mice

**Fig. 5:**
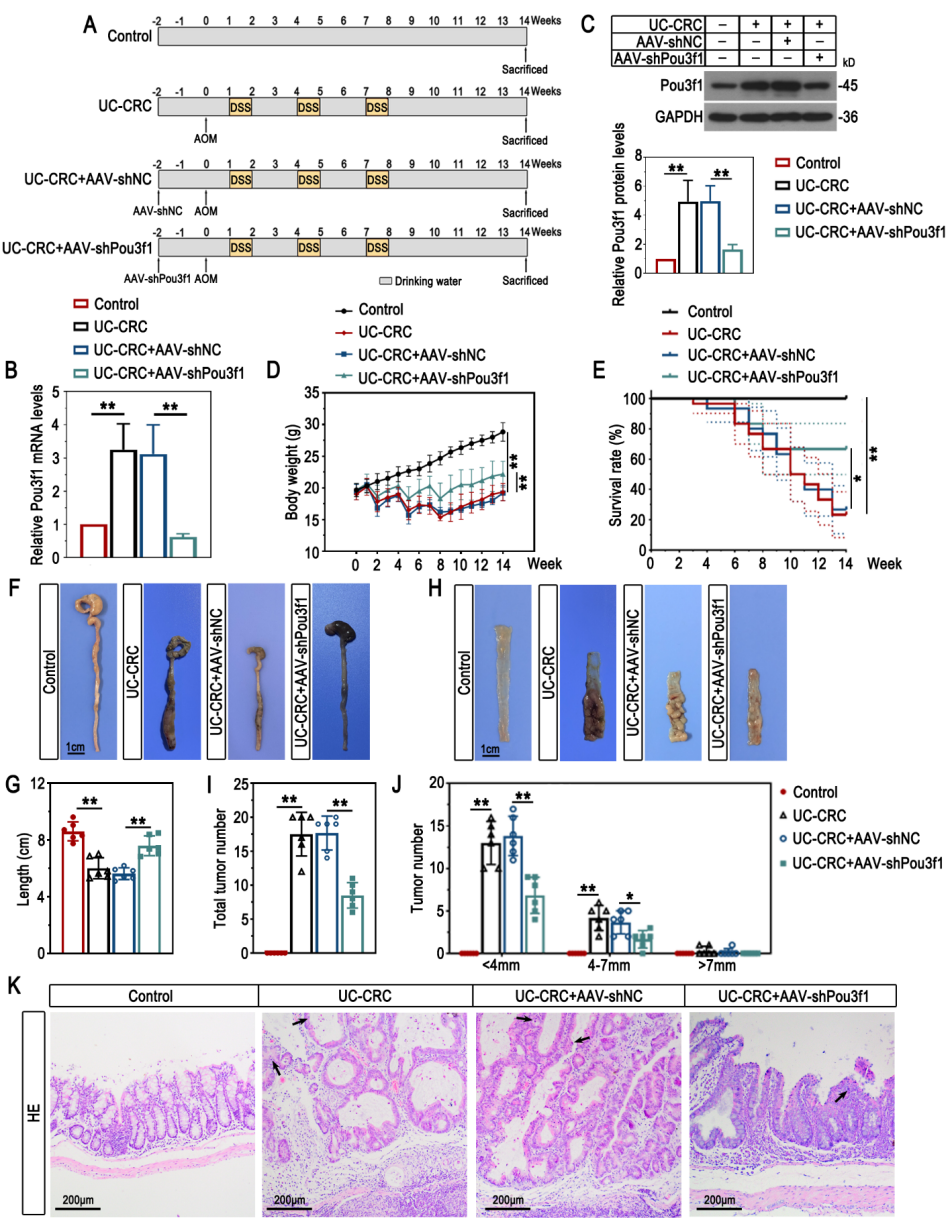
Pou3f1 knockdown protected against mucosal injury and colon tumorigenesis of UC-CRC mice. (A) The schematic diagram for animal model protocols. (B-C) The mRNA and protein levels of Pou3f1 in colons were measured by qPCR and Western blot. (D-E) The body weight and survival status of mice were recorded weekly. (F-G) The colon length was recorded. (H-J) Representative microscopic images of colon tumors. Total tumor number and the number of tumors with different diameters were counted. (K) Representative images of HE staining for colon tumors. The arrows indicated tumors. Data were from n = 6 mice per group for (B-D) and (F-K); n = 30 mice per group for (E). Values were mean ± SD. *, p <0.05; **, p < 0.01

AAV9-mediated Pou3f1 knockdown was performed for its loss-of-function study in vivo (Fig. 5B-C). During the modeling process, Pou3f1 depletion increased the body weight and survival ratio, as well as decreased the disease activity index in AOM/DSS-induced mice (Fig. 5D-E and Supplementary Fig. 4). Macroscopically, Pou3f1 knockdown increased colon length and attenuated colon injury in AOM/DSS-treated mice (Fig. 5F-G). The total tumor number and the number of tumors with different sizes were reduced by Pou3f1 inhibition (Fig. 5H-J). The histological results showed that inhibition of Pou3f1 mitigated crypt structure loss, inflammatory cell infiltration, and tumorigenesis in colons of AOM/DSS-induced mice (Fig. 5K). It indicated that Pou3f1 might drive the development of colitis-associated colorectal cancer.

### Pou3f1 knockdown inhibited the inflammation in colons of UC-CRC mice

**Fig. 6:**
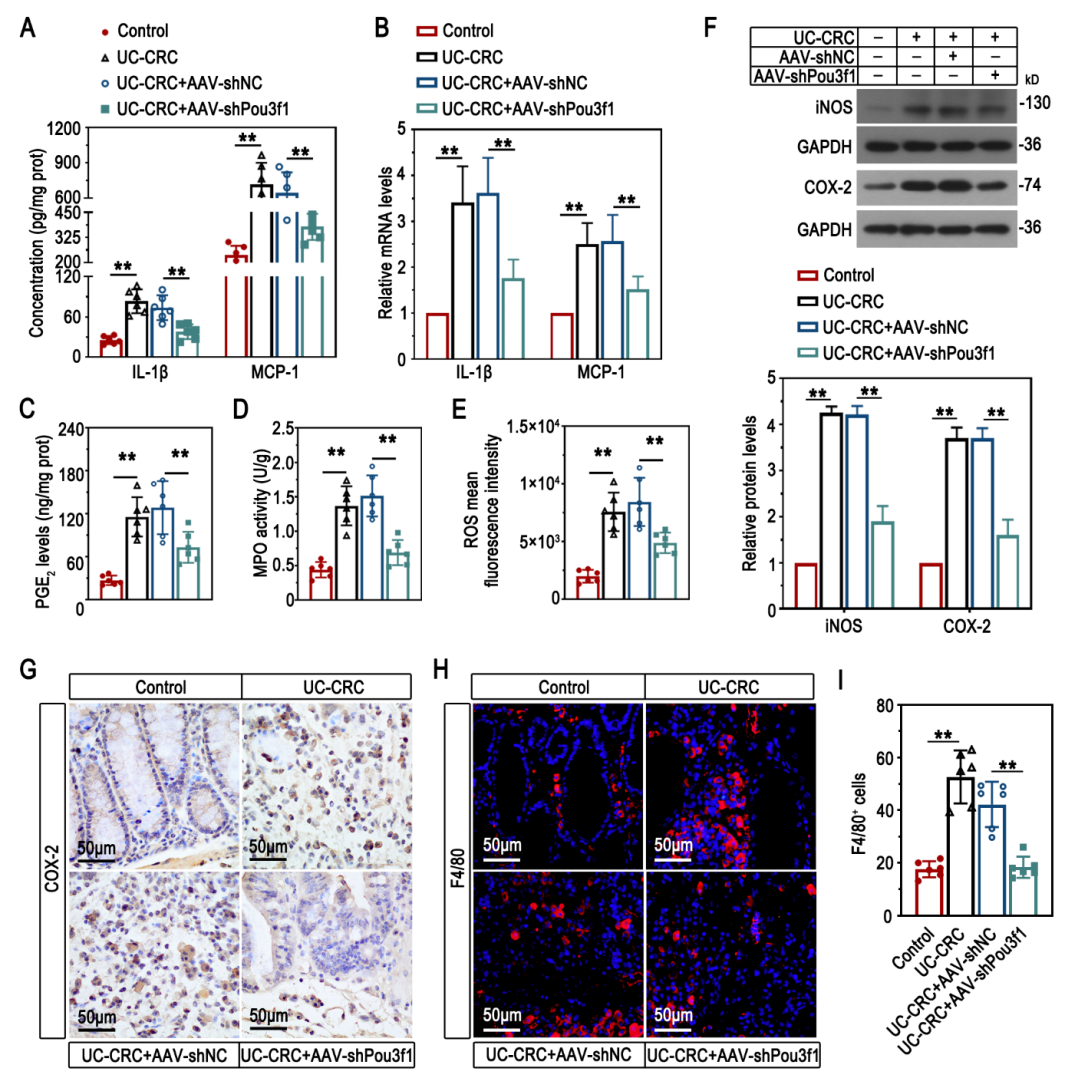
Pou3f1 knockdown inhibited inflammation in colons of UC-CRC mice. (A) The concentration of IL-1β and MCP-1 was measured using ELISA. (B) The relative mRNA levels of IL-1β and MCP-1 were determined by qPCR. (C-E) The PGE_2_ concentration, MPO activity, and ROS production were measured using commercial kits. (F) Western blot analysis for iNOS and COX-2 protein levels. (G) Representative immunohistochemical images of COX-2 in colons. (H-I) Representative immunofluorescent images of F4/80 (red) and quantification results. DAPI (blue) was used to stain the cell nuclei. Data were from n = 6 mice per group. Values were mean ± SD. **, p < 0.01

Chronic inflammation is regarded as a main contributor to colitis-associated colorectal cancer. Thus, we focused to unveil the role of Pou3f1 in regulating the inflammatory response in UC-CRC development. Pou3f1 knockdown reduced the concentration and mRNA expression of IL-1β and MCP-1 in colons of AOM/DSS-treated mice (Fig. 6A-B). The PGE_2_ level, MPO activity and ROS production in colons of UC-CRC were suppressed by AAV-shPou3f1 (Fig. 6C-E). AOM/DSS-induced iNOS and COX-2 was inhibited by Pou3f1 inhibition in colons (Fig. 6F-G). Furthermore, we demonstrated that knockdown of Pou3f1 caused a reduction of F4/80^+^ cells in UC-CRC mouse colons, indicating the promotive effect of Pou3f1 on macrophage abundance (Fig. 6H-I). Thus, the results suggested that Pou3f1 promoted inflammation and inflammatory cell infiltration in colitis-associated colorectal cancer.

### Pou3f1 knockdown affected cell proliferation and death in colon tumors of UC-CRC mice

**Fig. 7:**
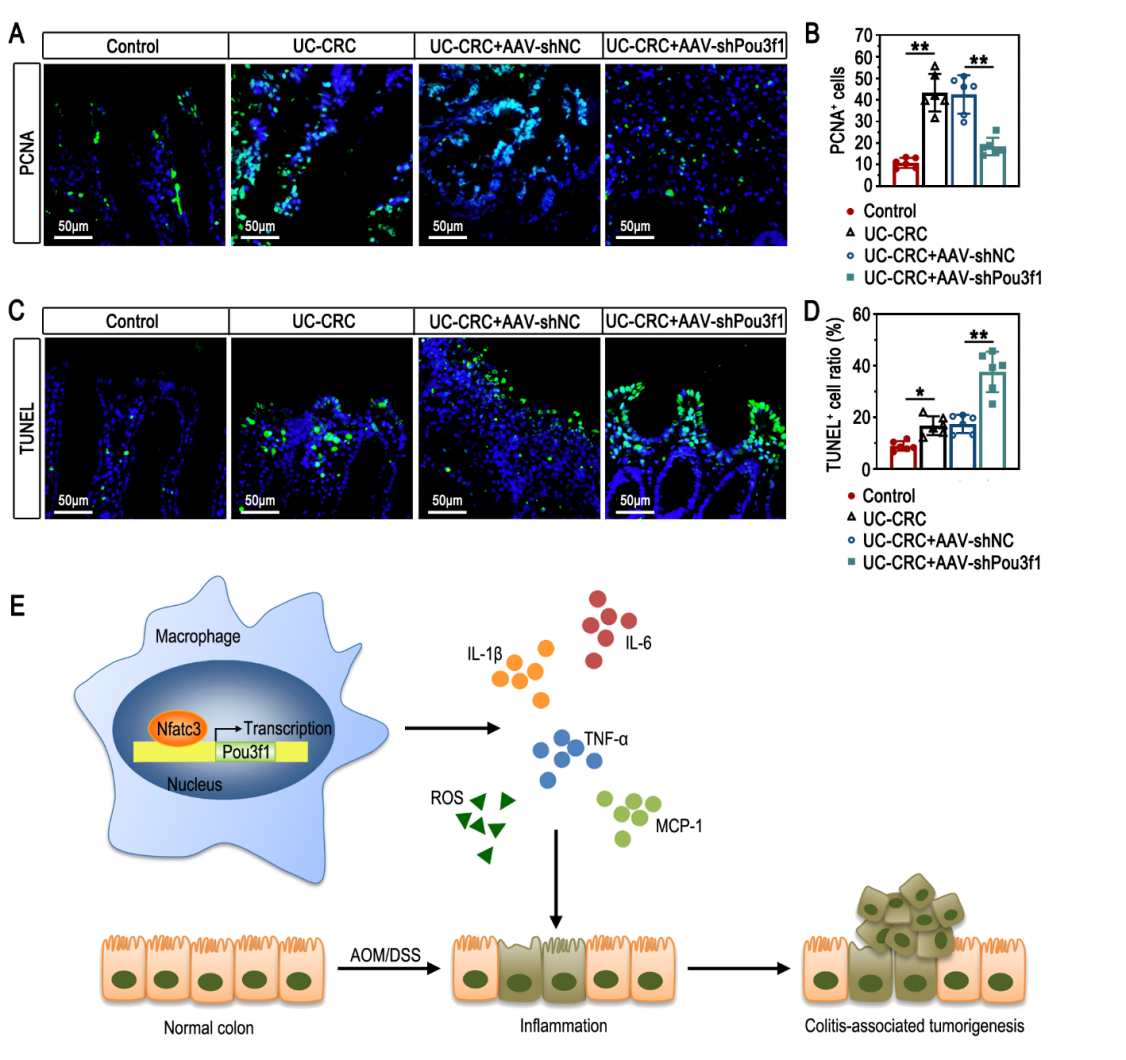
Pou3f1 knockdown affected cell proliferation and death in colon tumors of UC-CRC mice. (A-B) Representative immunofluorescent images and quantification results of PCNA (green). DAPI (blue) was used to stain the cell nuclei. (C-D) Representative images and quantification results of TUNEL-positive cells (green). DAPI (blue) was used to stain the cell nuclei. (E) The schematic diagram for the regulatory mechanism of Nfatc3-Pou3f1 in the development of UC-CRC. Data were from n = 6 mice per group. Values were mean ± SD. *, p < 0.05; **, p < 0.01

The epithelial inflammation is an essential initiator of tumorigenesis in UC-CRC. The expression of PCNA in colon epithelium of UC-CRC mice was inhibited by Pou3f1 knockdown (Fig. 7A-B). Results in Fig. 7C-D showed that Pou3f1 inhibition caused an increase of TUNEL-positive cells in colon epithelium of UC-CRC mice. These findings showed that Pou3f1 increased cell proliferation and reduced cell death in UC-CRC.

## Discussion

Chronic inflammation is a leading cause of colon carcinogenesis in inflammatory bowel diseases [27–29]. Herein, we demonstrated that knockdown of Pou3f1 suppressed inflammation and reduced colon tumorigenesis in UC-CRC. Pou3f1 was identified as a direct target of Nfatc3, and it mediated the proinflammatory effect of Nfatc3 in macrophages.

AOM is a chemical carcinogen by facilitating base mispairings, and the combined treatment of AOM and DSS induces inflammation and colon tumorigenesis [30, 31]. Our study demonstrated that the expression of Pou3f1 was increased in colons of AOM/DSS-induced UC-CRC mice. However, it is unclear that whether and how Pou3f1 mediates the tumorigenic process in UC-CRC. It is well-demonstrated that PGE_2_ and MPO are essential pro-oncogenic factors that activate carcinogenic signaling pathways and promote tumorigenesis in UC-CRC [32, 33]. The epithelial cell proliferation and death are basic factors in the intestinal tumorigenesis in response to inflammation [2]. PCNA is responsible for DNA synthesis in the nuclei, and it is commonly used as a marker for cell proliferation [34]. Inducing epithelial cell death during tumorigenic process is critical for the therapy or prevention of UC-CRC [35]. The regulation on TUNEL-positive cells seems important in the control of tumor progression of UC-CRC [36]. In line with previous studies, we demonstrated that Pou3f1 inhibition reduced pro-oncogenic cytokine production, suppressed cell proliferation and increased cell death in UC-CRC [22, 37].

Macrophages in the intestinal tract maintain mucosal homeostasis and carcinogenesis [8, 38]. Bader et al. demonstrated that lack of macrophages inhibited inflammation and tumor growth in AOM/DSS-induced mice [39]. This work showed an infiltration of a number of macrophages into the colons of UC-CRC. The infiltrated macrophages secret proinflammatory factors (including TNF-α, IL-1β, IL-6, ROS and MCP-1) to initiate tumorigenesis [2, 10, 40]. COX-2 and iNOS are key mediators of inflammation, and they contribute to the tumor formation in colons [41]. Our study showed that knockdown of Pou3f1 abrogated inflammatory mediator secretion in macrophages, resulting in the inhibition of tumor growth in UC-CRC. However, there is an inconsistency between ours and the previous results. Fionda et al. indicated that Pou3f1 depletion increased the intracellular ROS levels in non-small-cell lung carcinoma cells in the presence of doxorubicin [22]. A possible reason for this discrepancy is that macrophages and cancer cells are heterogeneous.

We further investigated the underlying mechanism of Pou3f1 in UC-CRC progression. Our results showed that Pou3f1 physically interacted with Nfatc3 by ChIP assay. Then, the luciferase reporter assay confirmed three binding sites of Nfatc3 in the sequence of Pou3f1 promoter and demonstrated the positive regulation of Nfatc3 on Pou3f1 transcriptional activity. However, other potential binding sites in the Pou3f1 promoter by which Nfatc3 regulates Pou3f1 expression remain to be further investigated. In addition to the transcriptional regulation by Nfatc3, Barral et al. demonstrated that Pou3f1 was directly targeted by the transcription factor Nanog [42]. The position of the binding sites of Nanog is far from that of Nfatc3 in the Pou3f1 promoter, thus we speculate that Nfatc3 and Nanog may not competitively regulate the transcription of Pou3f1. So far, there is no evidence to show the co-regulation by other factors through the binding sites of Nfatc3 in the Pou3f1 promotor that were confirmed in this study. Of course, we don’t rule out this possibility. It needs more studies to prove. Furthermore, the previous study revealed that Nfatc3 cooperated with another transcription factor MyoD to regulate myogenin expression [43]. Wollebo et al. suggested that Nfatc3 and NF-κB p65 bound with the KB element to cooperatively activate JCV transcription [44]. Nevertheless, whether these factors involved in the regulation of Nfatc3 on Pou3f1 is still unknown and deserves further exploration in our further researches.

This work still has several limitations. For example, Jensen et al. have demonstrated that Pou3f1 is important for neurogenesis [21]. The nervous system is required for controlling tumor growth and metastasis, and neurogenesis is an independent indicator of poor clinical outcomes in CRC [45–47]. However, the involvement of Pou3f1 in the neurogenesis of UC-CRC is not yet stated. More investigations are required in the future for a comprehensive understanding of Pou3f1’s role in UC-CRC.

## Conclusion

The present work suggests that Pou3f1 is a direct transcriptional target of Nfatc3, and contributes to macrophage-related inflammation and tumorigenesis in UC-CRC (Fig. 7E). It may provide a novel and potential therapeutic target for UC-CRC.

## Electronic supplementary material

Below is the link to the electronic supplementary material.

Supplementary Material 1

Supplementary Material 2

## Data Availability

The datasets used and/or analyzed during the current study are available from the corresponding author on reasonable request.

## List of abbreviations

AOM: Azoxymethane
BMDMs: Bone marrow-derived macrophages
ChIP: Chromatin immunoprecipitation
DSS: Dextran sulfate sodium
ELISA: Enzyme-linked immunosorbent assay
HE: Hematoxylin and eosin
IL-1β: Interleukin-1β
IL-6: Interleukin-6
MCP-1: Monocyte chemotactic protein-1
MPO: Myeloperoxidase
Nfatc3: Nuclear factor of activated T cells 3
PCNA: Proliferating Cell Nuclear Antigen
PGE_2_: Prostaglandin E2
Pou3f1: POU class 3 homeobox 1
qPCR: Quantitative real-time PCR
ROS: Reactive oxygen species
TNF-α: Tumor necrosis factor-α
UC-CRC: Ulcerative colitis-associated colorectal cancer
