# Supplementary material for: Pou3f1 mediates the effect of Nfatc3 on ulcerative colitis-associated colorectal cancer by regulating inflammation
Source: Cell Mol Biol Lett. 2022 Sep 5;27:75. doi: 10.1186/s11658-022-00374-0 (PMC9446766; doi:10.1186/s11658-022-00374-0)
Supplement: Supplementary file 1 — Supplementary Material 1 [file 11658_2022_374_MOESM1_ESM.docx]

**Supplementary files**


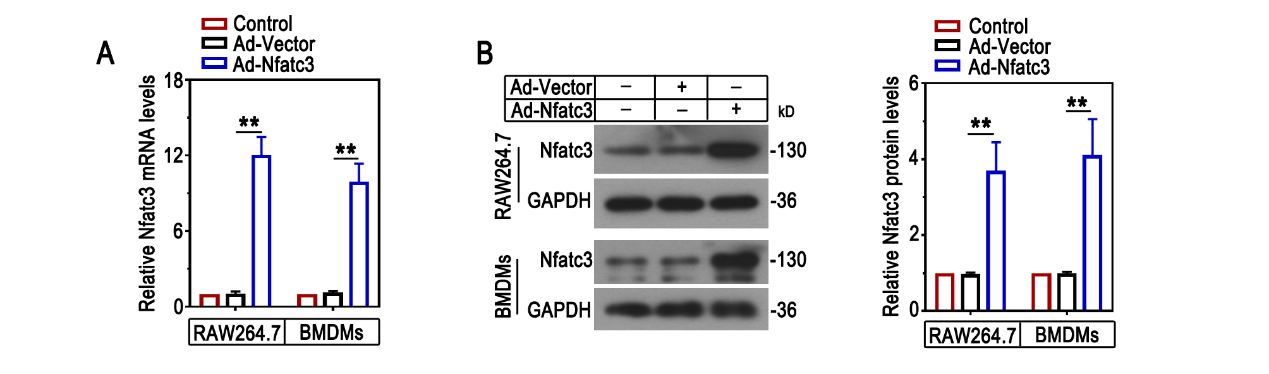


Supplementary Figure 1. The infection efficiency for the adenoviral vectors carrying Nfatc3 in macrophages. (A) qPCR analysis for the mRNA level of Nfatc3 in RAW264.7 cells and BMDMs. (B) Western blot analysis and quantification results for the protein level of Nfatc3 in RAW264.7 cells and BMDMs. n=3. Values were mean ± SD. **, p<0.01.


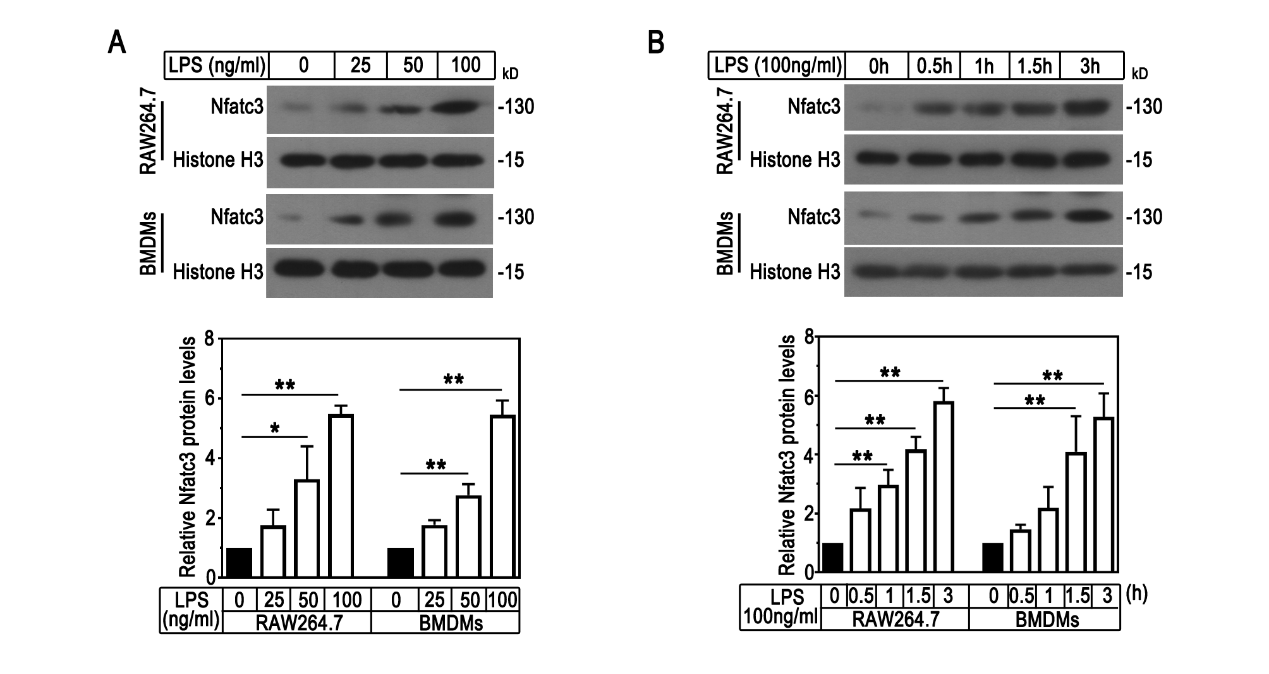


Supplementary Figure 2. The expression of Nfatc3 in LPS-induced macrophages. (A) Western blot analysis and quantification results for the protein level of Nfatc3 in RAW264.7 cells and BMDMs in response to LPS with different concentrations. (B) Western blot analysis and quantification results for the protein level of Nfatc3 in RAW264.7 cells and BMDMs in response to LPS at the indicated time. n=3. Values were mean ± SD. *, p<0.05; **, p<0.01.


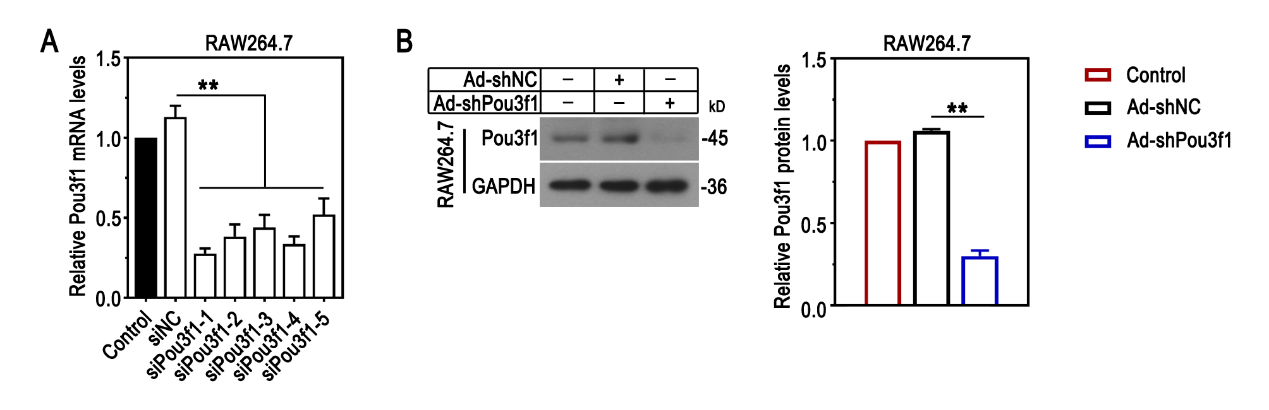


Supplementary Figure 3. The infection efficiency for the adenoviral vectors carrying shPou3f1 in RAW264.7 cells. (A) The interference sequence of Pouf3f1 was transiently transfected into cells. qPCR analysis was performed to determine the mRNA level of Pou3f1. (B) The adenoviral vector carrying shPou3f1 was infected into cells. Western blot analysis was used to examine the protein level of Pou3f1. n=3. Values were mean ± SD. **, p<0.01.


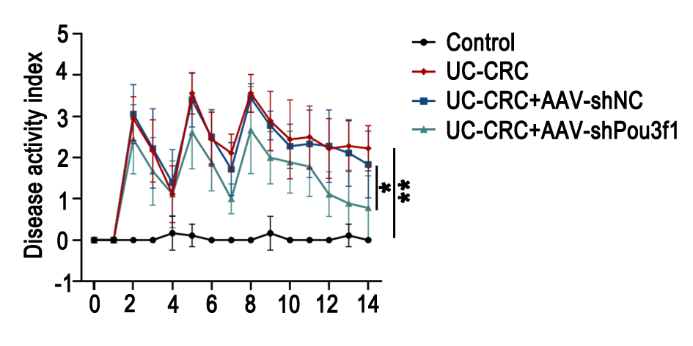


Supplementary Figure 4. Pou3f1 knockdown inhibited the disease activity index in UC-CRC mice. Data were from n=6 mice per group. Values were mean ± SD. *, p<0.05; **, p<0.01.
