# Supplementary material for: Pou3f1 mediates the effect of Nfatc3 on ulcerative colitis-associated colorectal cancer by regulating inflammation
Source: Cell Mol Biol Lett. 2022 Sep 5;27:75. doi: 10.1186/s11658-022-00374-0 (PMC9446766; doi:10.1186/s11658-022-00374-0)
Supplement: Supplementary file 2 — Supplementary Material 2 [file 11658_2022_374_MOESM2_ESM.pptx]

## Slide 1
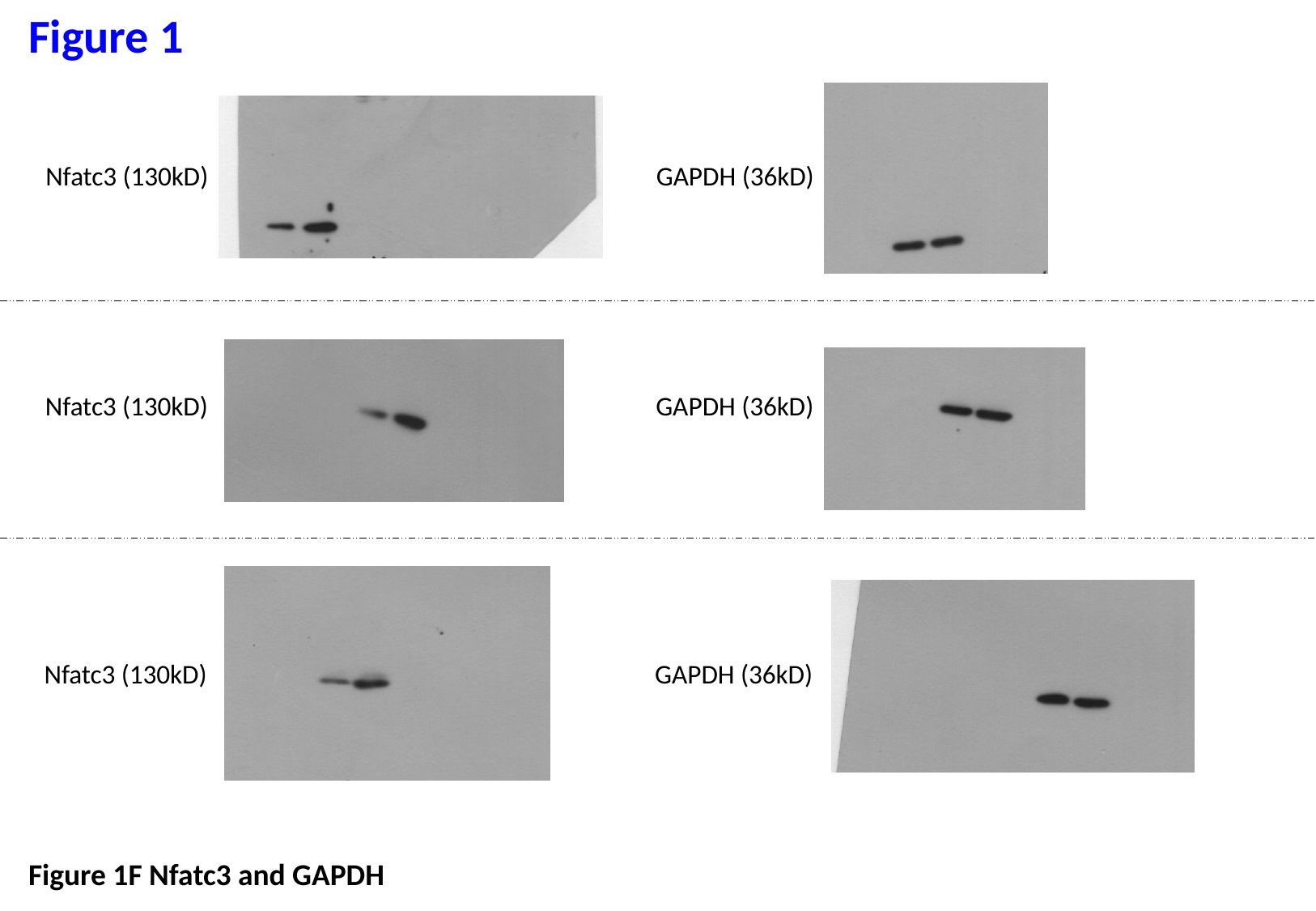

Figure 1
GAPDH (36kD)
Nfatc3 (130kD)
GAPDH (36kD)
Nfatc3 (130kD)
GAPDH (36kD)
Nfatc3 (130kD)
Figure 1F Nfatc3 and GAPDH

## Slide 2
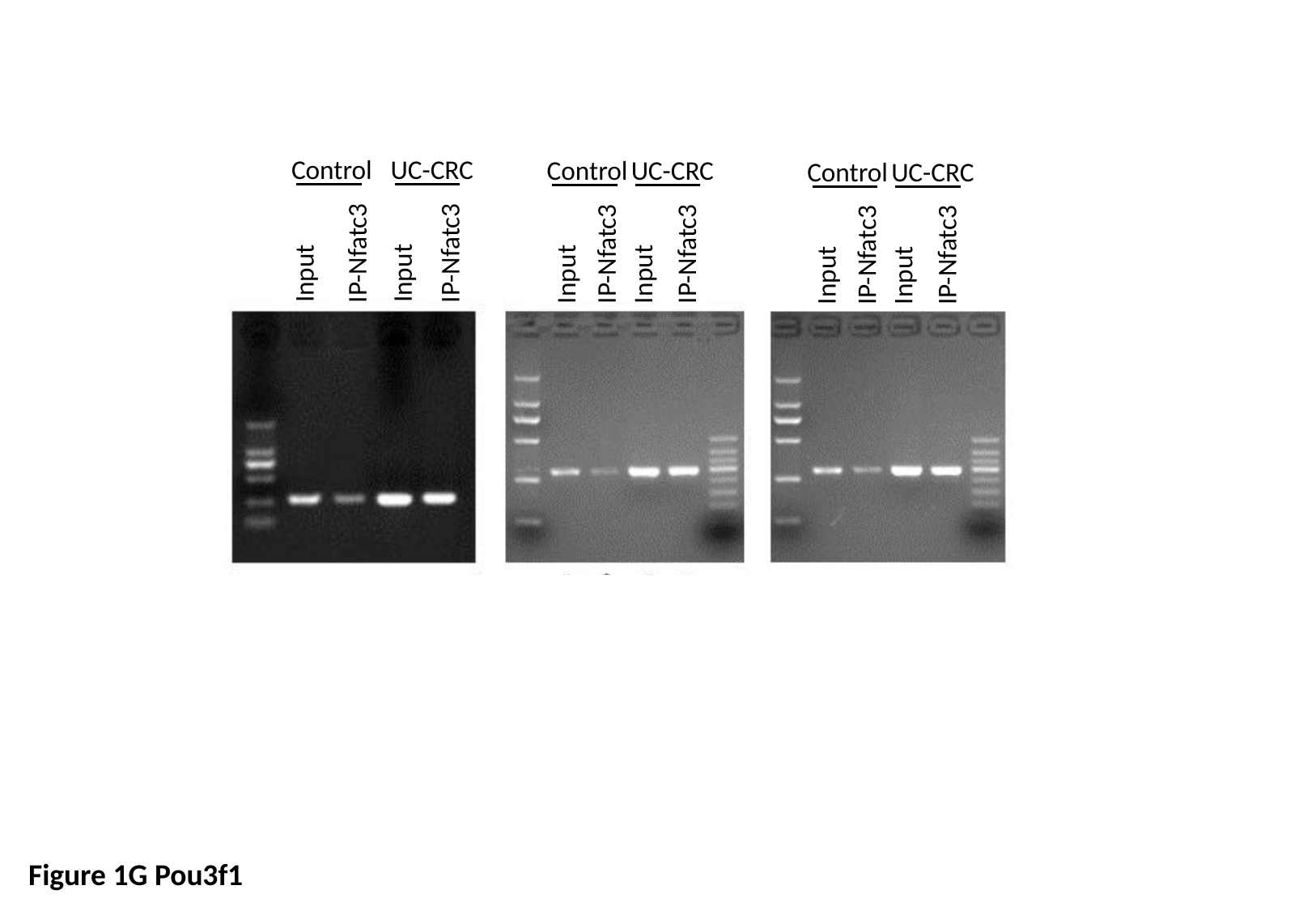

UC-CRC
Control
IP-Nfatc3
IP-Nfatc3
Input
Input
UC-CRC
Control
IP-Nfatc3
IP-Nfatc3
Input
Input
UC-CRC
Control
IP-Nfatc3
IP-Nfatc3
Input
Input
Figure 1G Pou3f1

## Slide 3
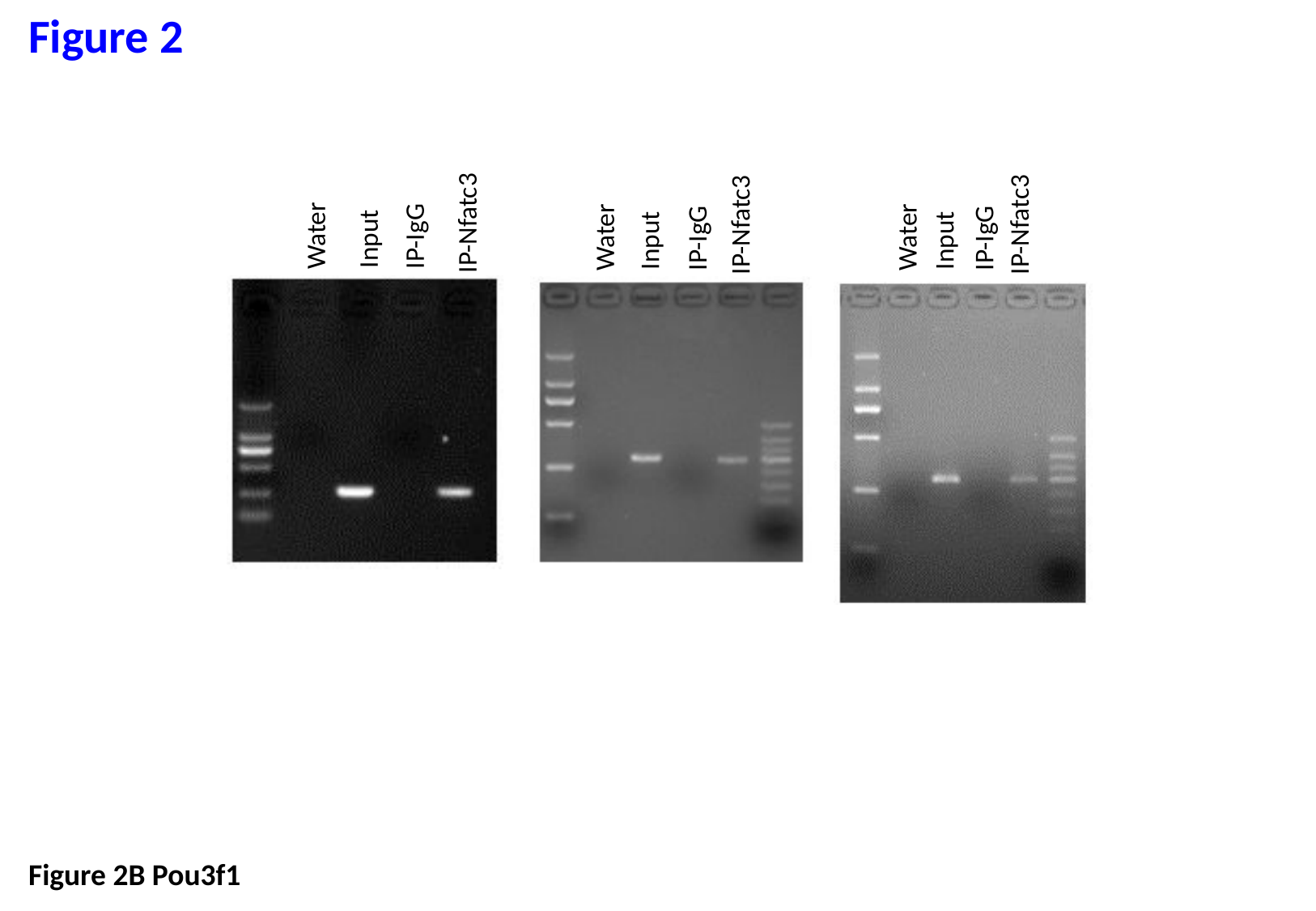

Figure 2
IP-Nfatc3
Water
IP-IgG
Input
IP-Nfatc3
Water
IP-IgG
Input
IP-Nfatc3
Water
IP-IgG
Input
Figure 2B Pou3f1

## Slide 4
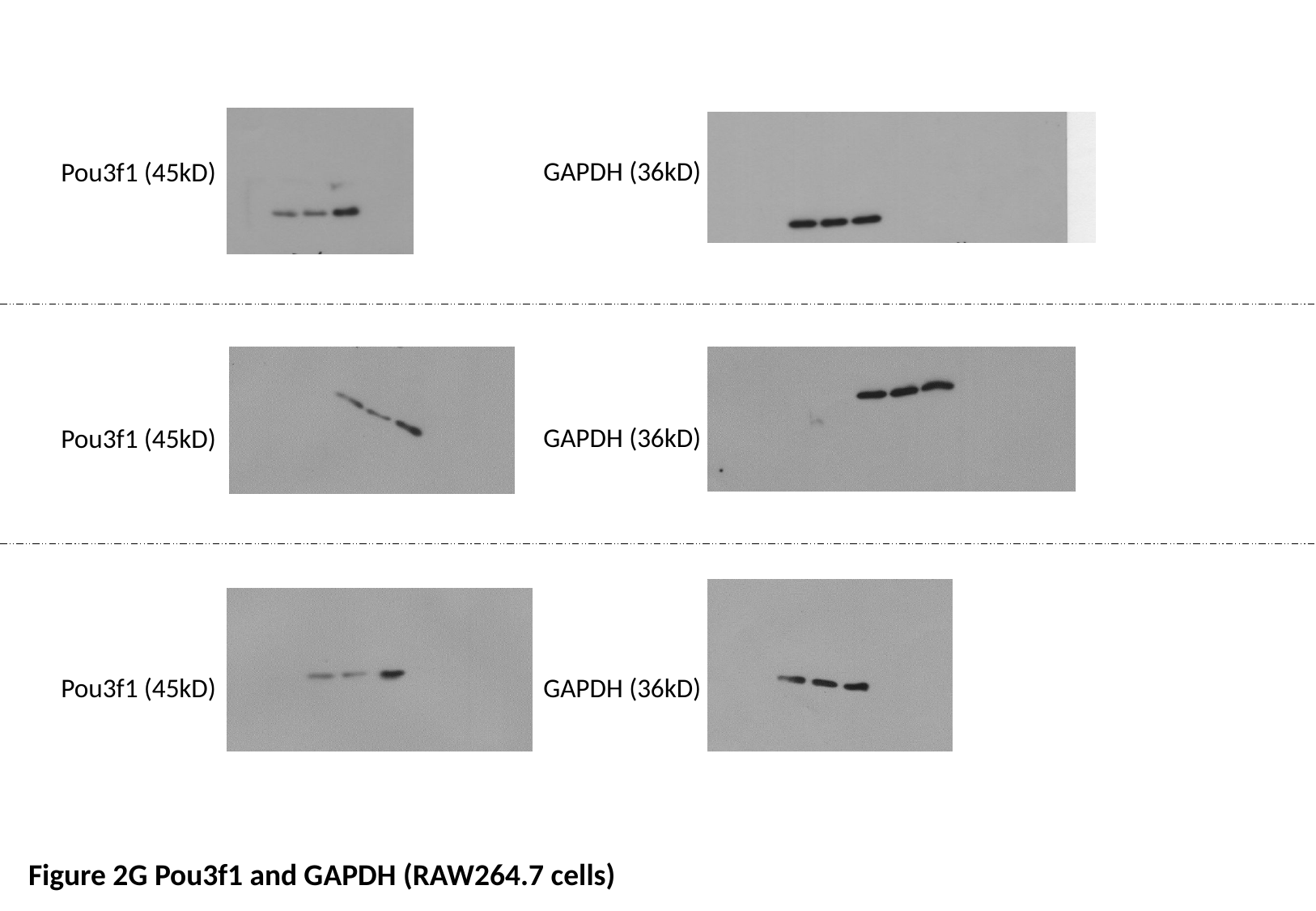

GAPDH (36kD)
Pou3f1 (45kD)
GAPDH (36kD)
Pou3f1 (45kD)
GAPDH (36kD)
Pou3f1 (45kD)
Figure 2G Pou3f1 and GAPDH (RAW264.7 cells)

## Slide 5
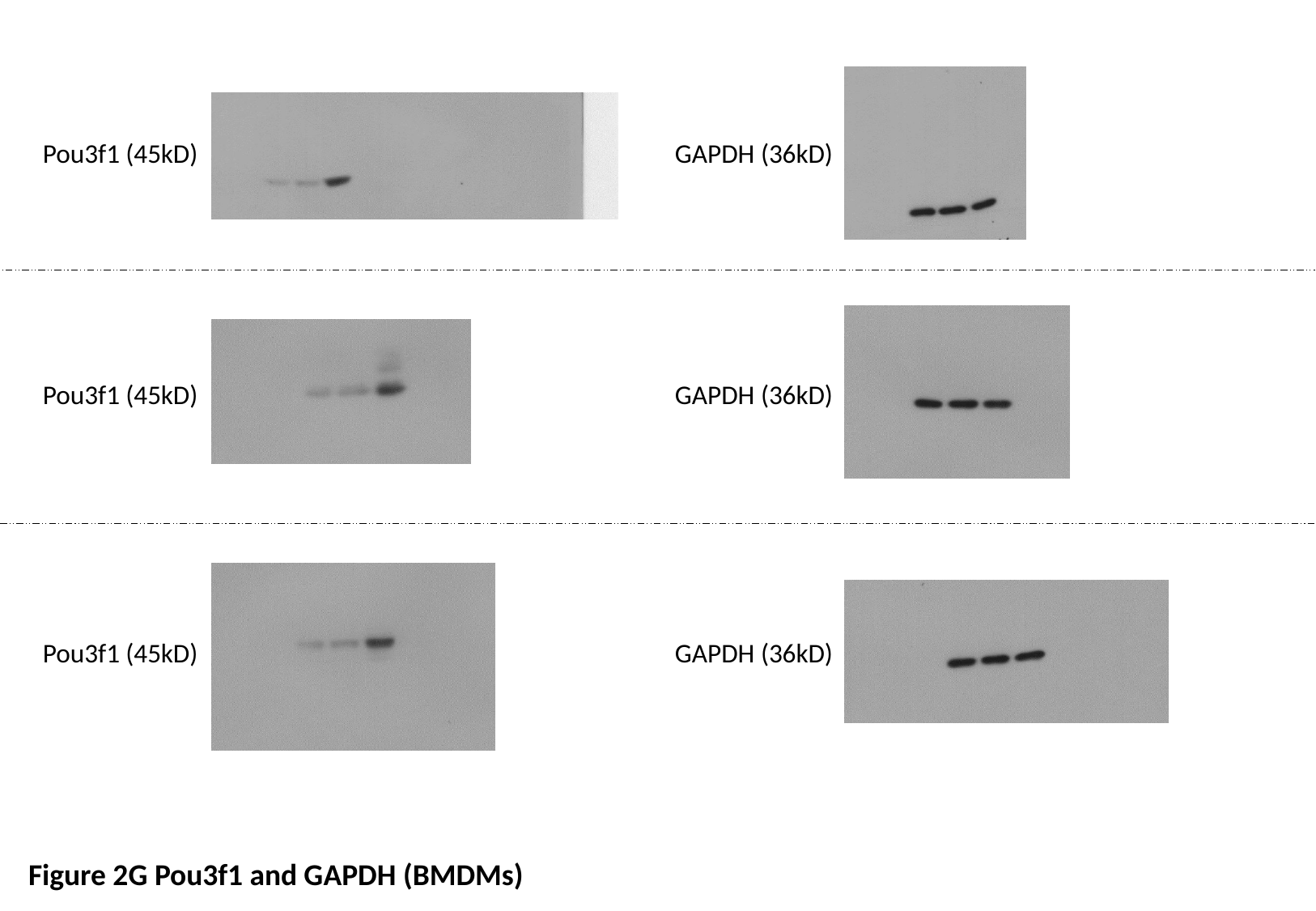

Pou3f1 (45kD)
GAPDH (36kD)
Pou3f1 (45kD)
GAPDH (36kD)
Pou3f1 (45kD)
GAPDH (36kD)
Figure 2G Pou3f1 and GAPDH (BMDMs)

## Slide 6
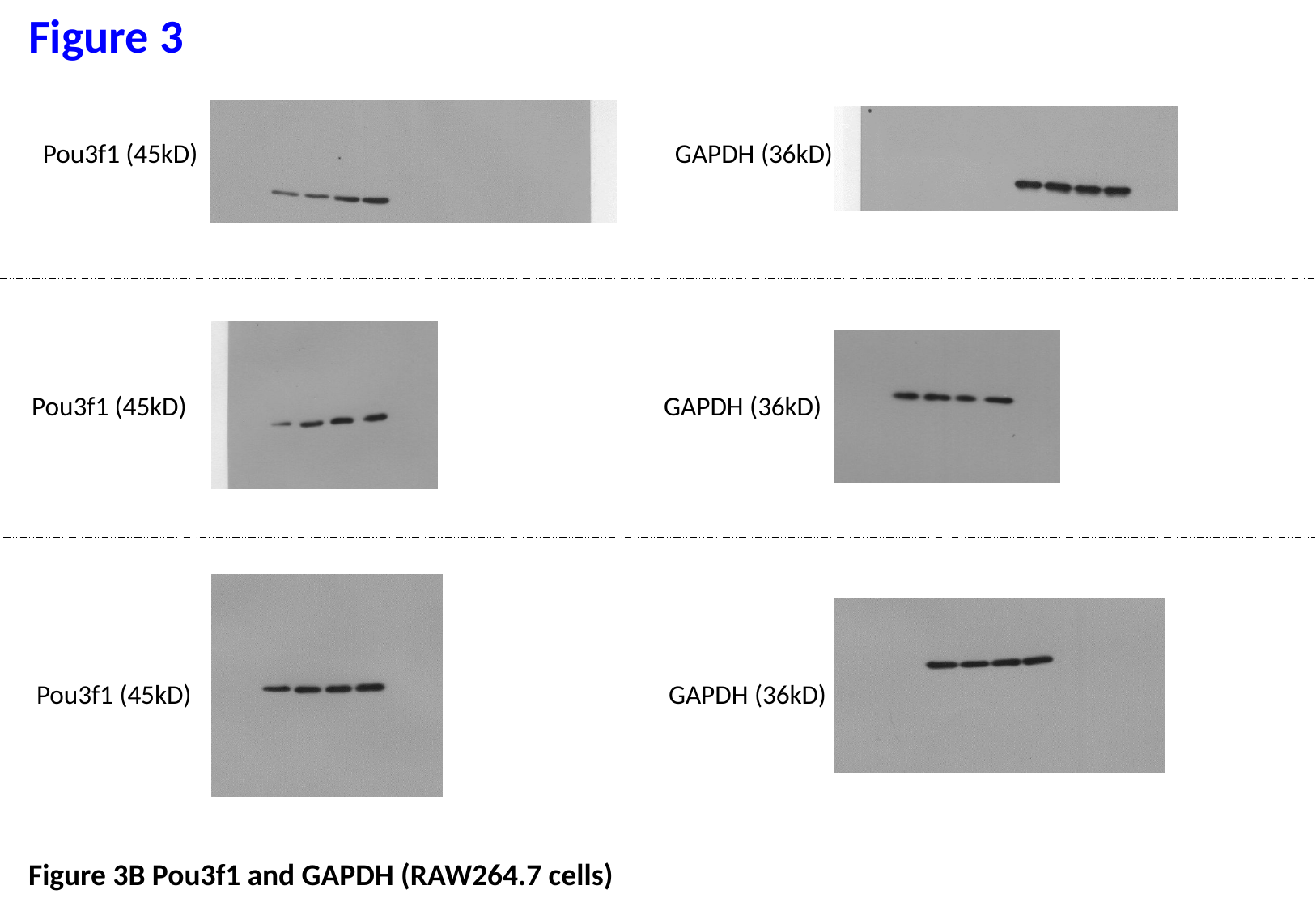

Figure 3
Pou3f1 (45kD)
GAPDH (36kD)
Pou3f1 (45kD)
GAPDH (36kD)
Pou3f1 (45kD)
GAPDH (36kD)
Figure 3B Pou3f1 and GAPDH (RAW264.7 cells)

## Slide 7
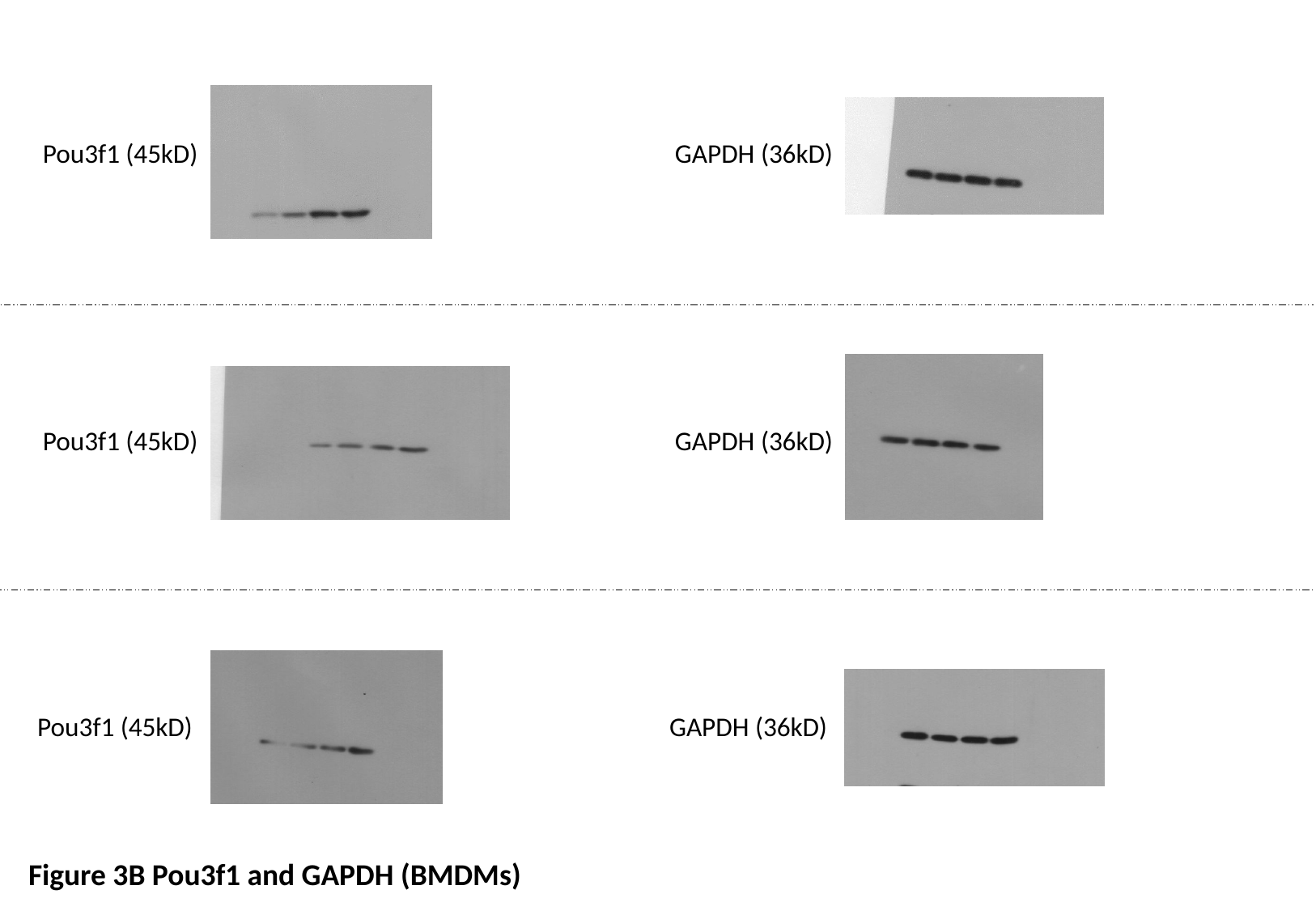

Pou3f1 (45kD)
GAPDH (36kD)
Pou3f1 (45kD)
GAPDH (36kD)
Pou3f1 (45kD)
GAPDH (36kD)
Figure 3B Pou3f1 and GAPDH (BMDMs)

## Slide 8
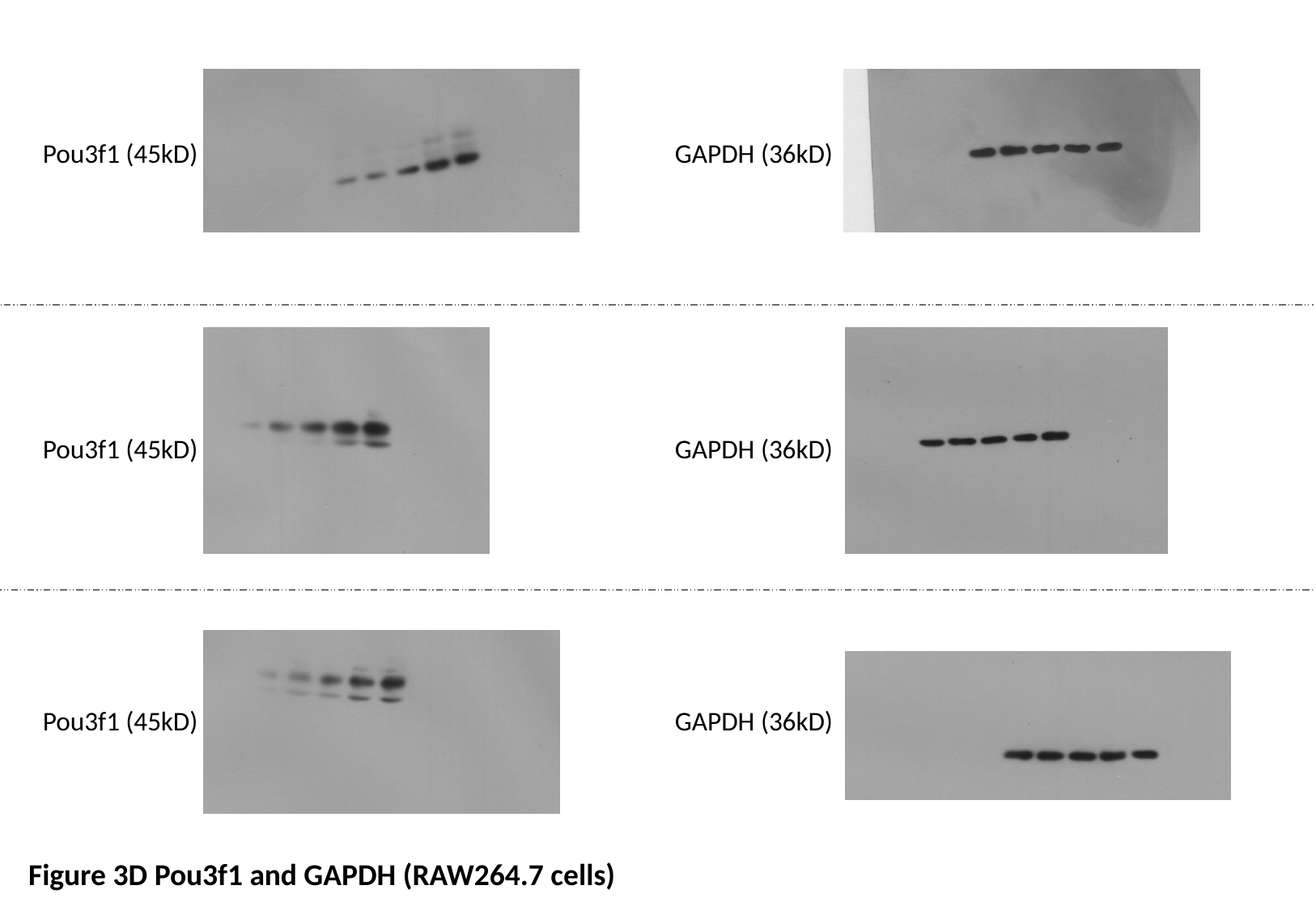

Pou3f1 (45kD)
GAPDH (36kD)
Pou3f1 (45kD)
GAPDH (36kD)
Pou3f1 (45kD)
GAPDH (36kD)
Figure 3D Pou3f1 and GAPDH (RAW264.7 cells)

## Slide 9
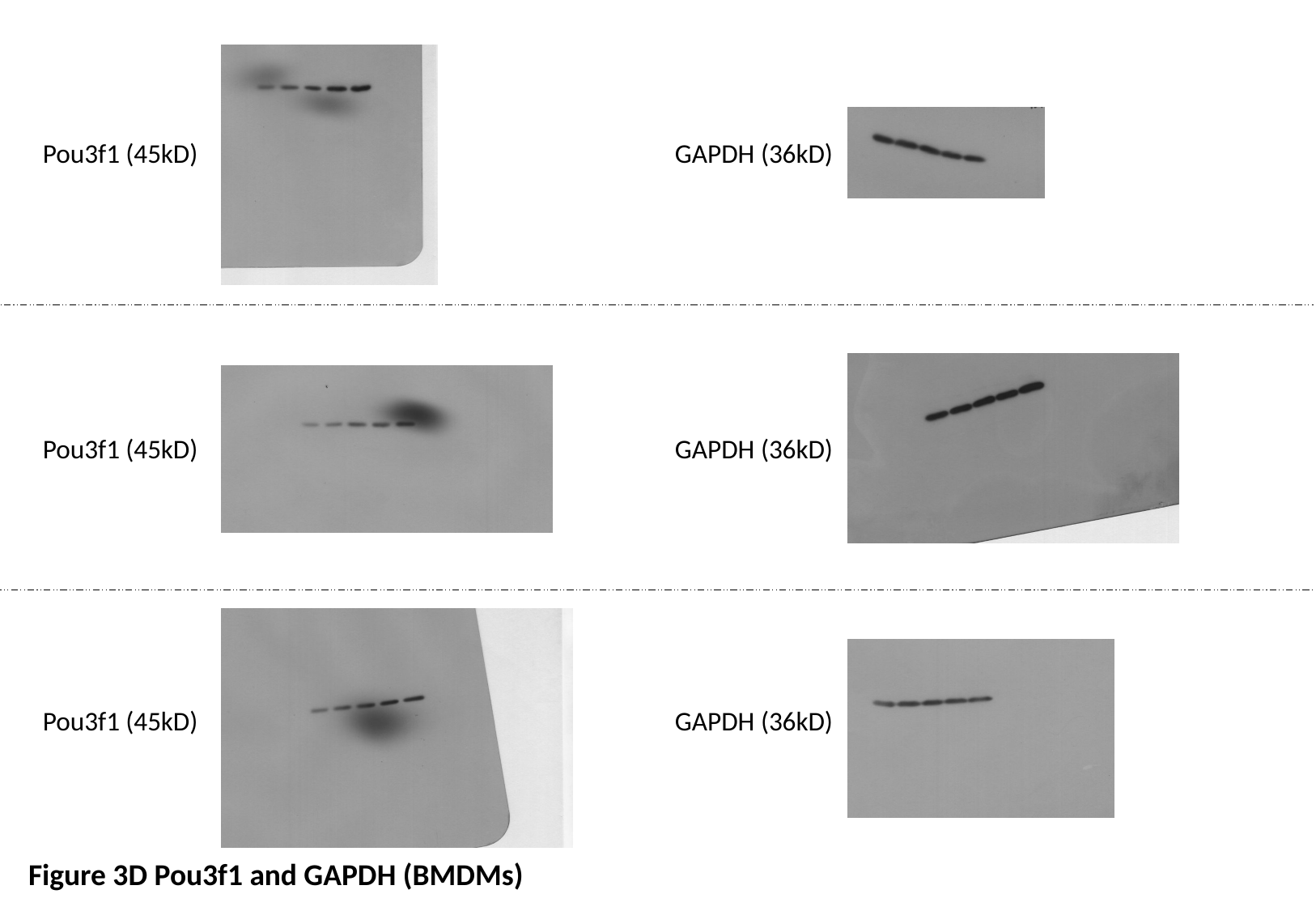

Pou3f1 (45kD)
GAPDH (36kD)
Pou3f1 (45kD)
GAPDH (36kD)
Pou3f1 (45kD)
GAPDH (36kD)
Figure 3D Pou3f1 and GAPDH (BMDMs)

## Slide 10
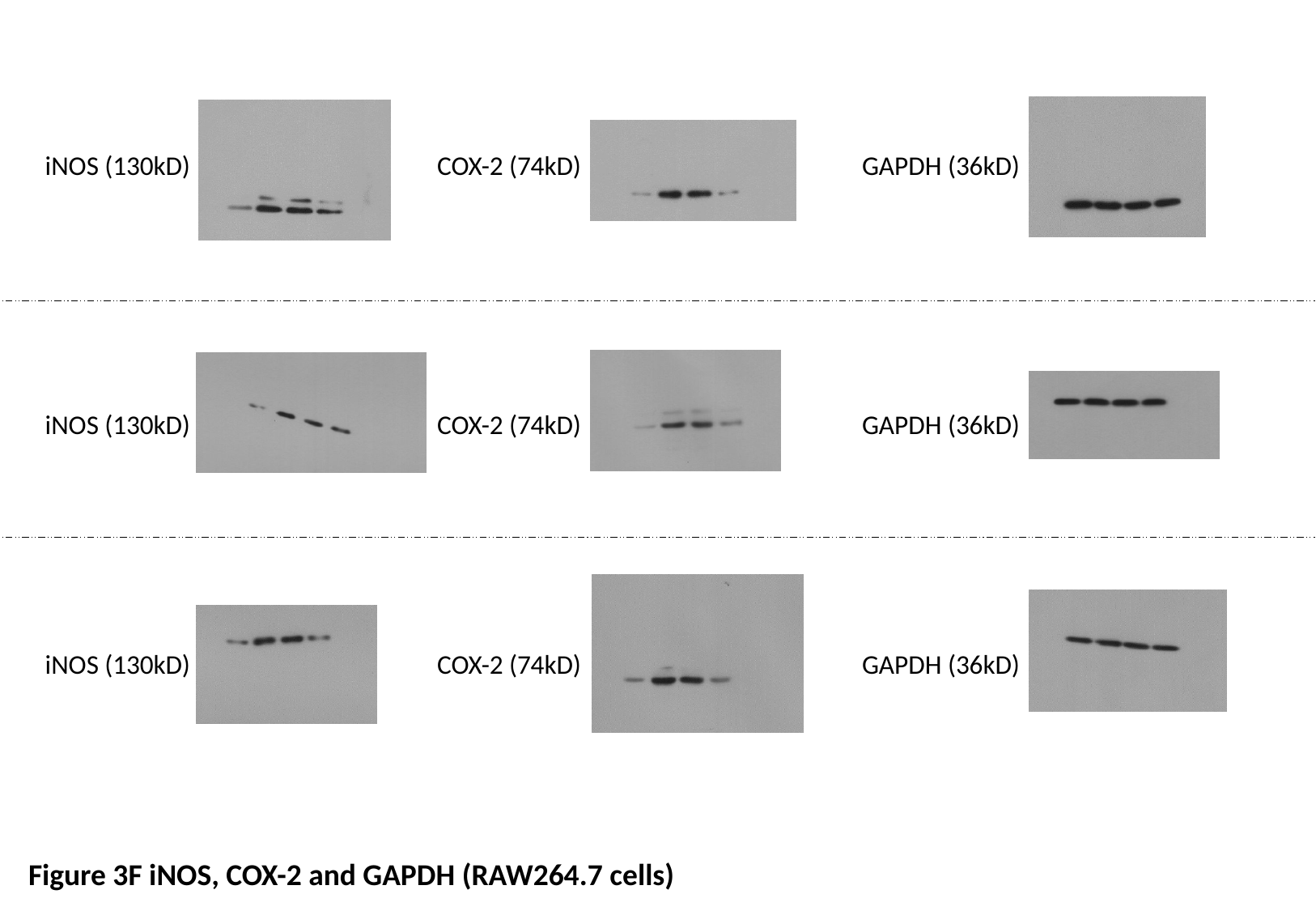

iNOS (130kD)
COX-2 (74kD)
GAPDH (36kD)
iNOS (130kD)
COX-2 (74kD)
GAPDH (36kD)
iNOS (130kD)
COX-2 (74kD)
GAPDH (36kD)
Figure 3F iNOS, COX-2 and GAPDH (RAW264.7 cells)

## Slide 11
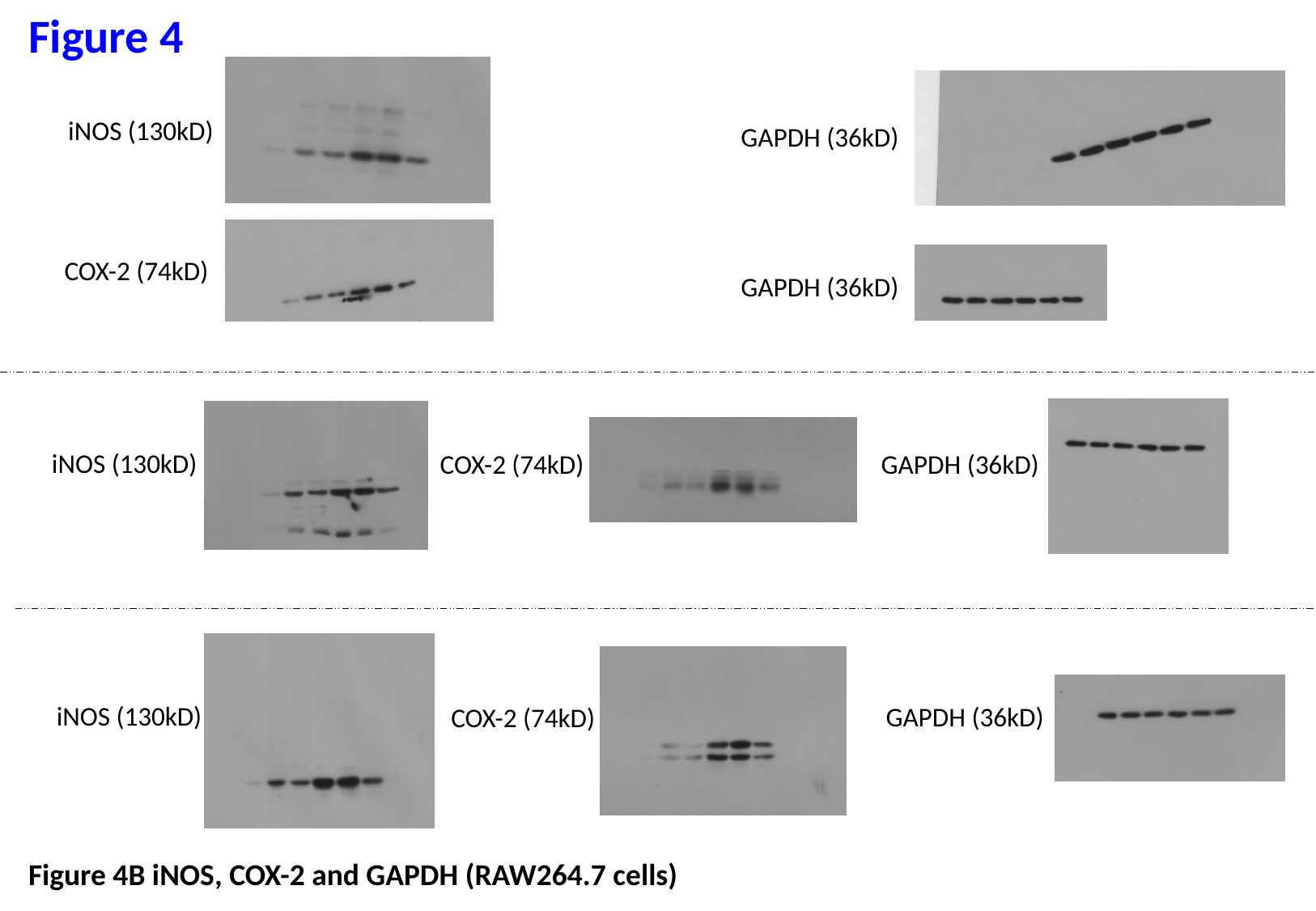

Figure 4
iNOS (130kD)
GAPDH (36kD)
COX-2 (74kD)
GAPDH (36kD)
iNOS (130kD)
COX-2 (74kD)
GAPDH (36kD)
iNOS (130kD)
GAPDH (36kD)
COX-2 (74kD)
Figure 4B iNOS, COX-2 and GAPDH (RAW264.7 cells)

## Slide 12
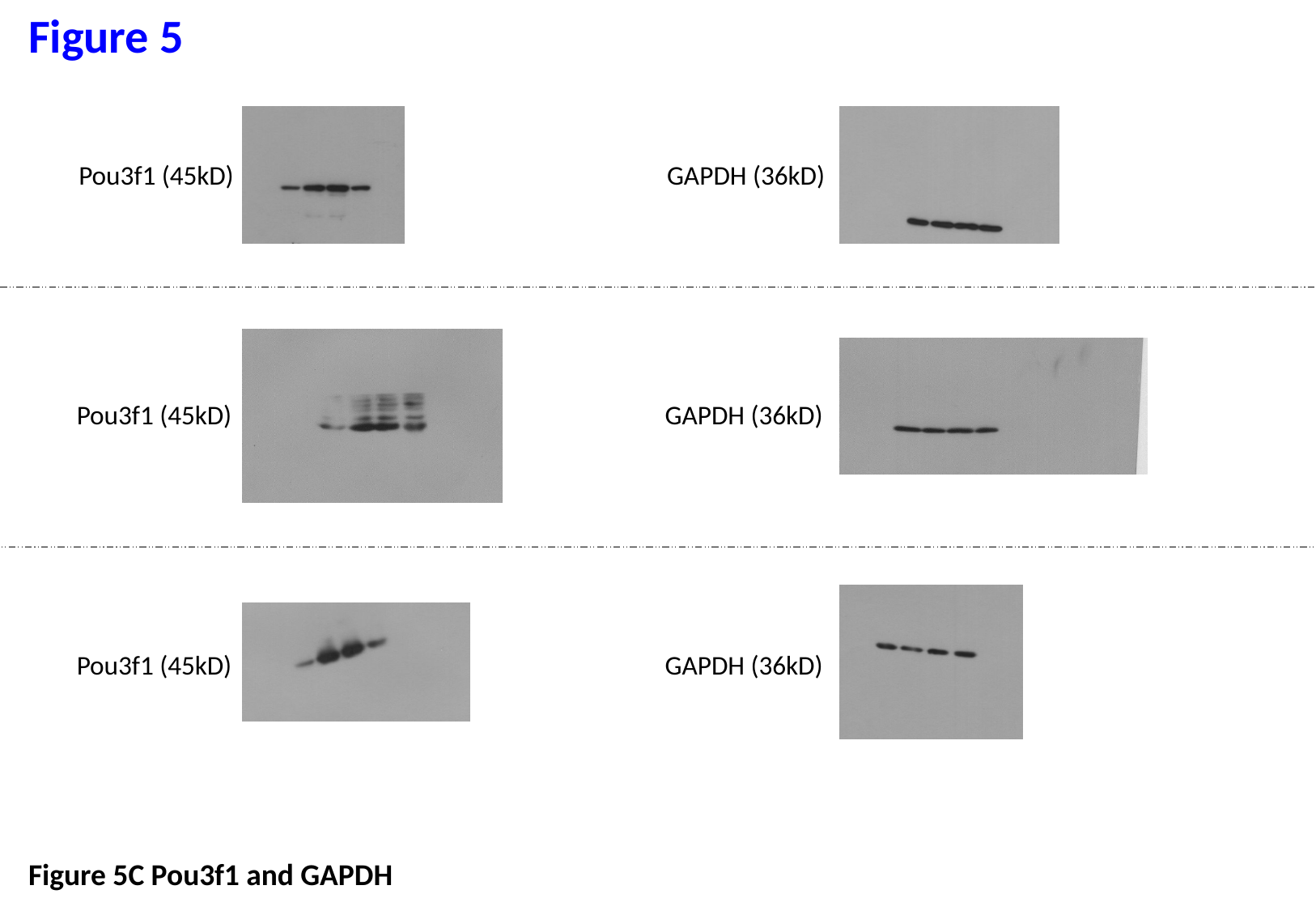

Figure 5
GAPDH (36kD)
Pou3f1 (45kD)
GAPDH (36kD)
Pou3f1 (45kD)
GAPDH (36kD)
Pou3f1 (45kD)
Figure 5C Pou3f1 and GAPDH

## Slide 13
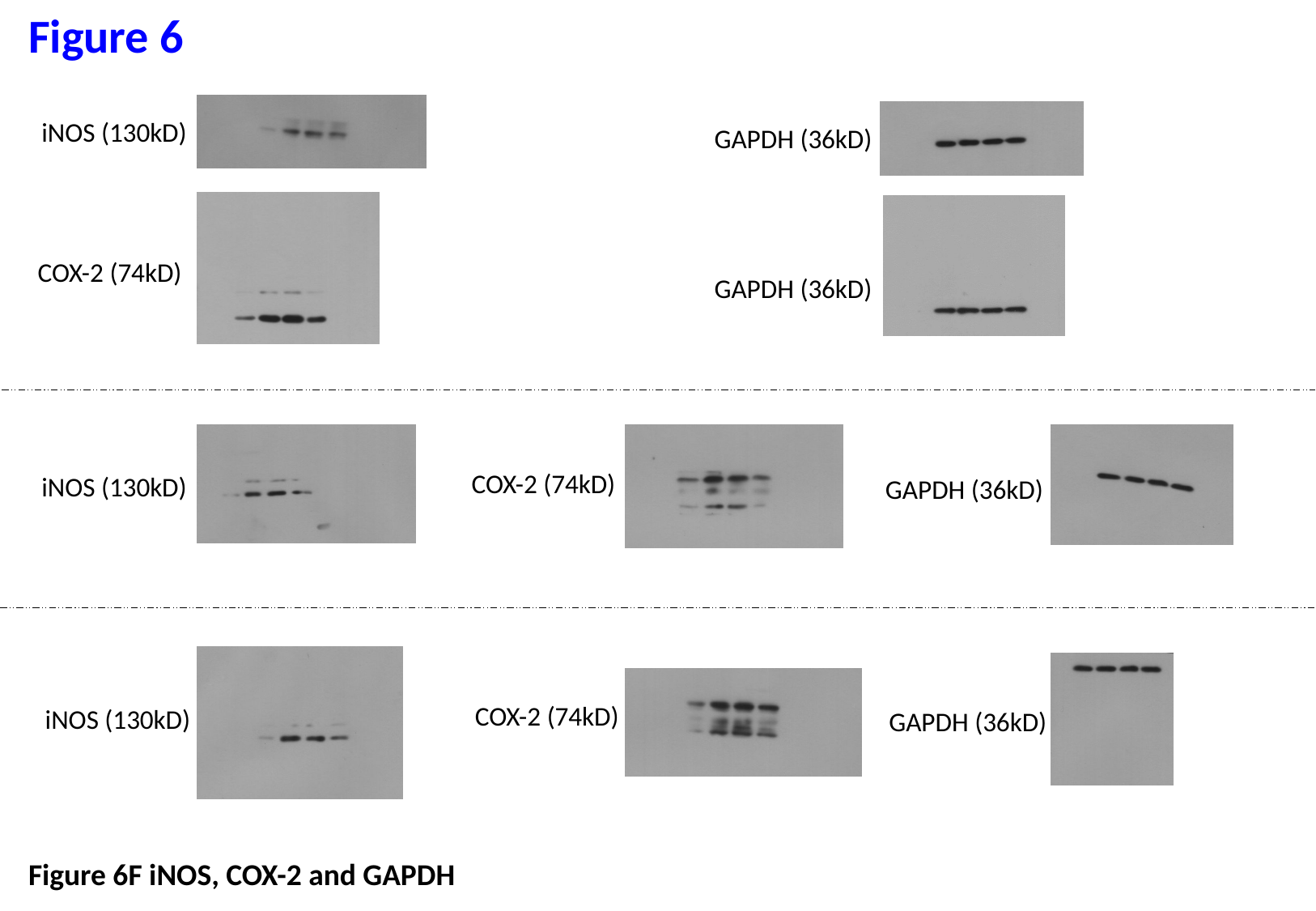

Figure 6
iNOS (130kD)
GAPDH (36kD)
COX-2 (74kD)
GAPDH (36kD)
COX-2 (74kD)
iNOS (130kD)
GAPDH (36kD)
COX-2 (74kD)
iNOS (130kD)
GAPDH (36kD)
Figure 6F iNOS, COX-2 and GAPDH

## Slide 14
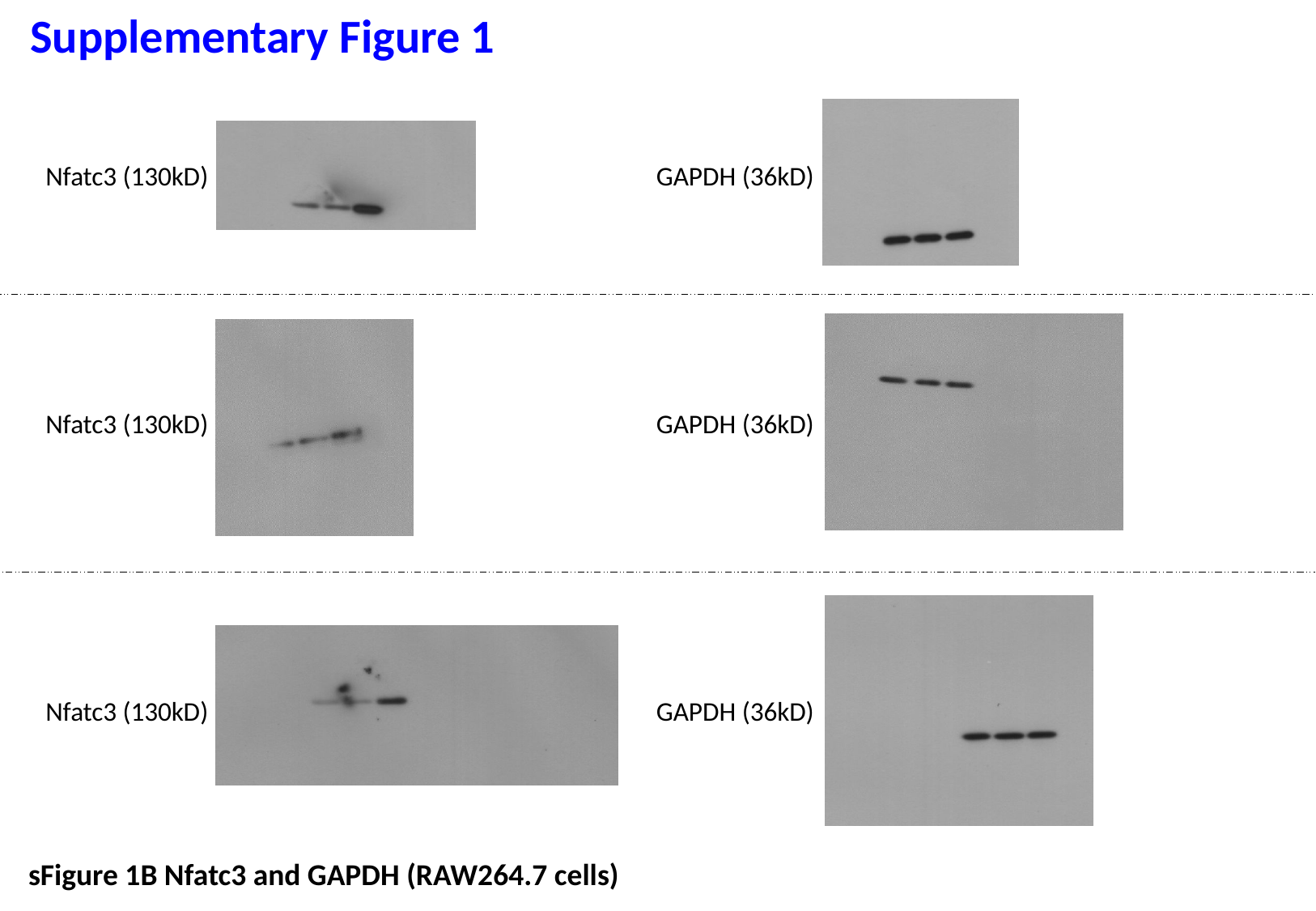

Supplementary Figure 1
GAPDH (36kD)
Nfatc3 (130kD)
GAPDH (36kD)
Nfatc3 (130kD)
GAPDH (36kD)
Nfatc3 (130kD)
sFigure 1B Nfatc3 and GAPDH (RAW264.7 cells)

## Slide 15
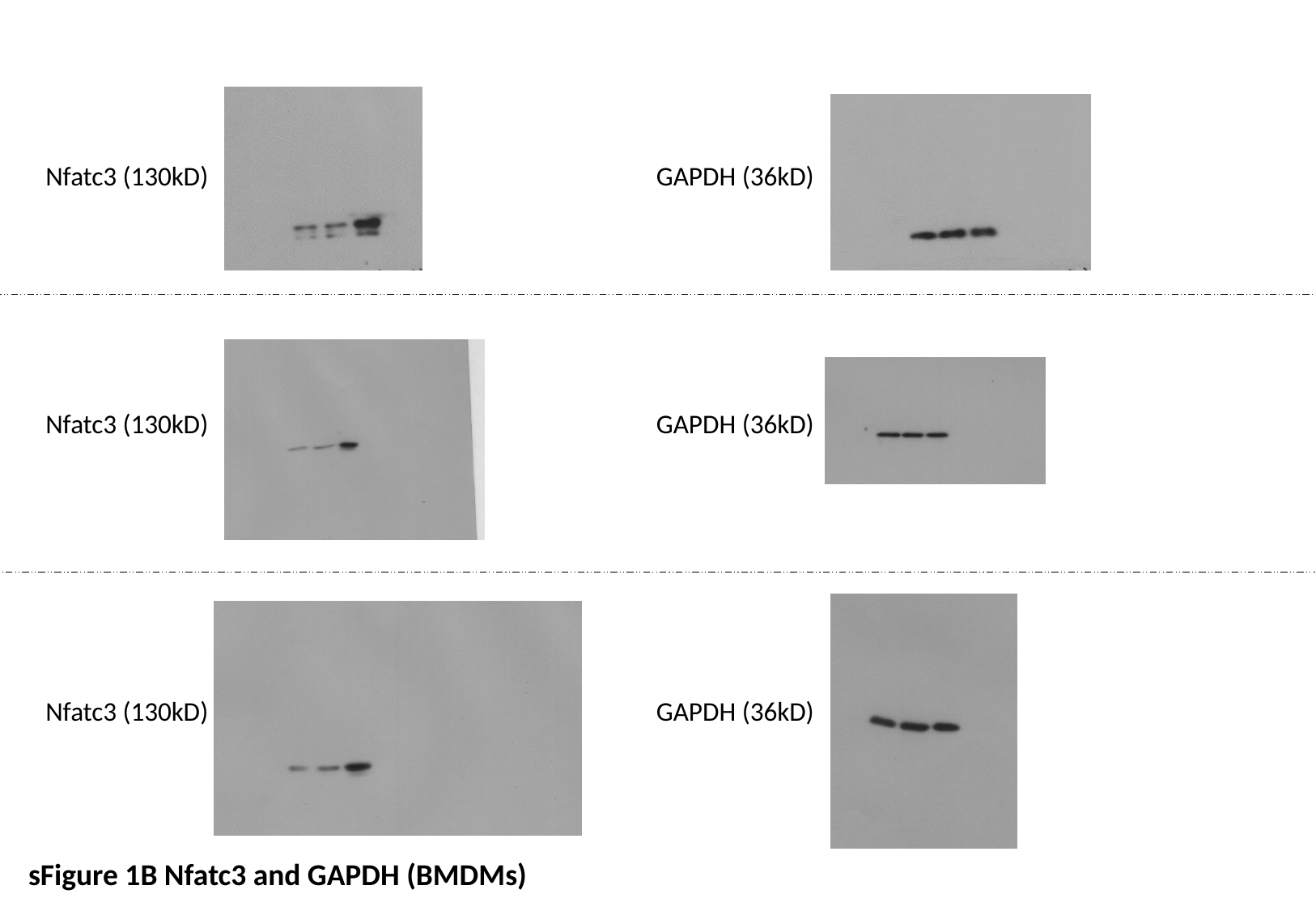

GAPDH (36kD)
Nfatc3 (130kD)
GAPDH (36kD)
Nfatc3 (130kD)
GAPDH (36kD)
Nfatc3 (130kD)
sFigure 1B Nfatc3 and GAPDH (BMDMs)

## Slide 16
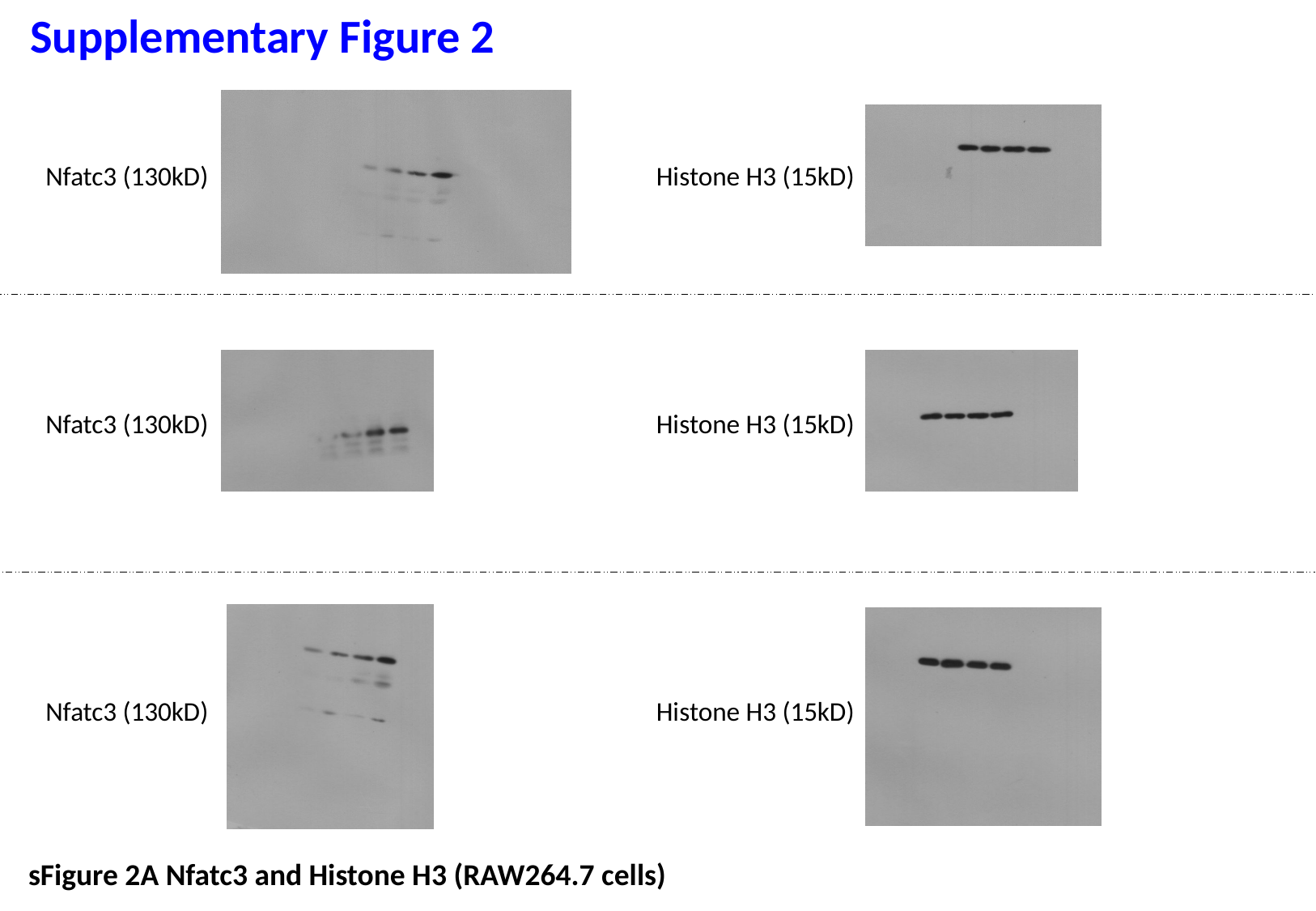

Supplementary Figure 2
Histone H3 (15kD)
Nfatc3 (130kD)
Histone H3 (15kD)
Nfatc3 (130kD)
Histone H3 (15kD)
Nfatc3 (130kD)
sFigure 2A Nfatc3 and Histone H3 (RAW264.7 cells)

## Slide 17
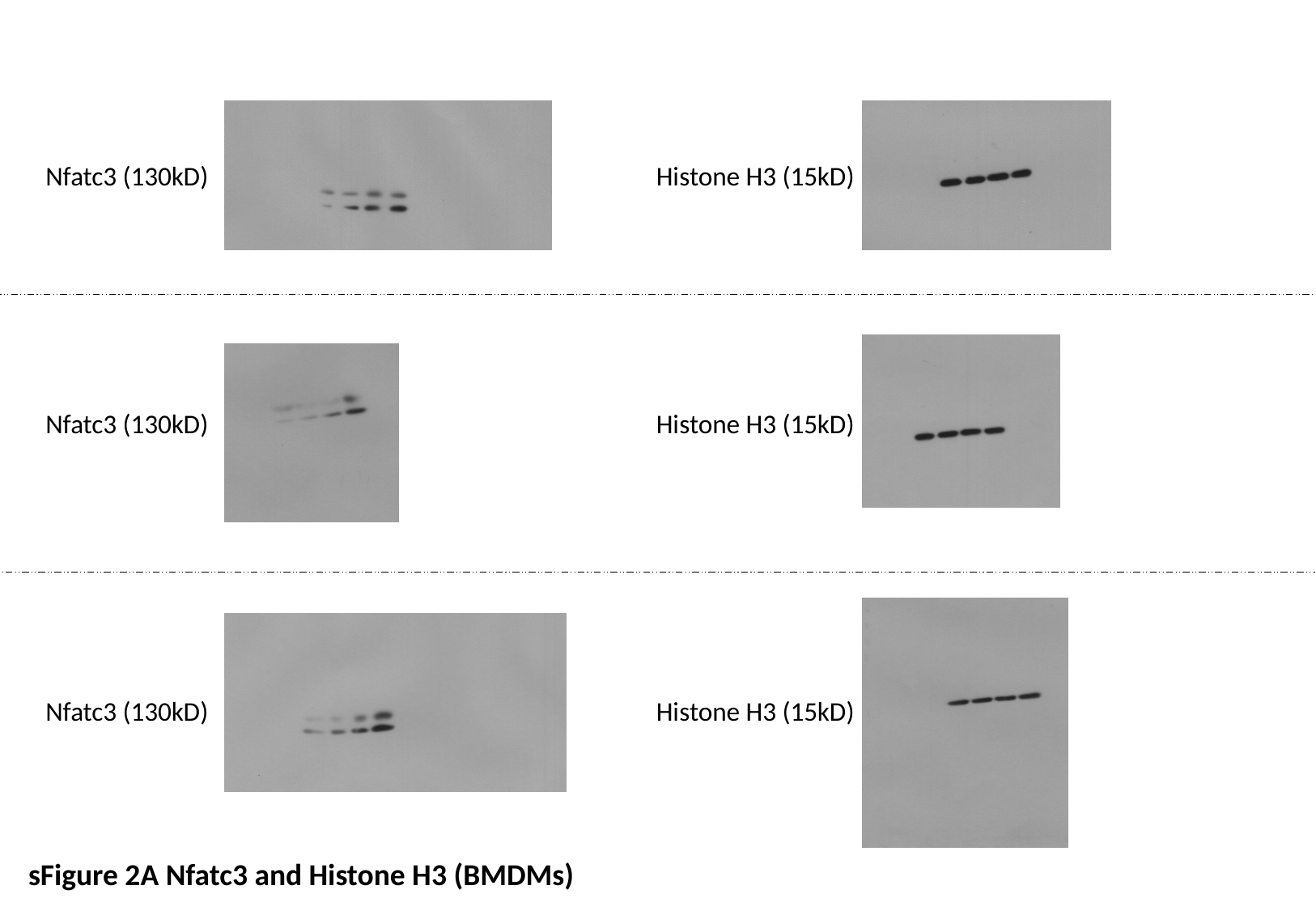

Histone H3 (15kD)
Nfatc3 (130kD)
Histone H3 (15kD)
Nfatc3 (130kD)
Histone H3 (15kD)
Nfatc3 (130kD)
sFigure 2A Nfatc3 and Histone H3 (BMDMs)

## Slide 18
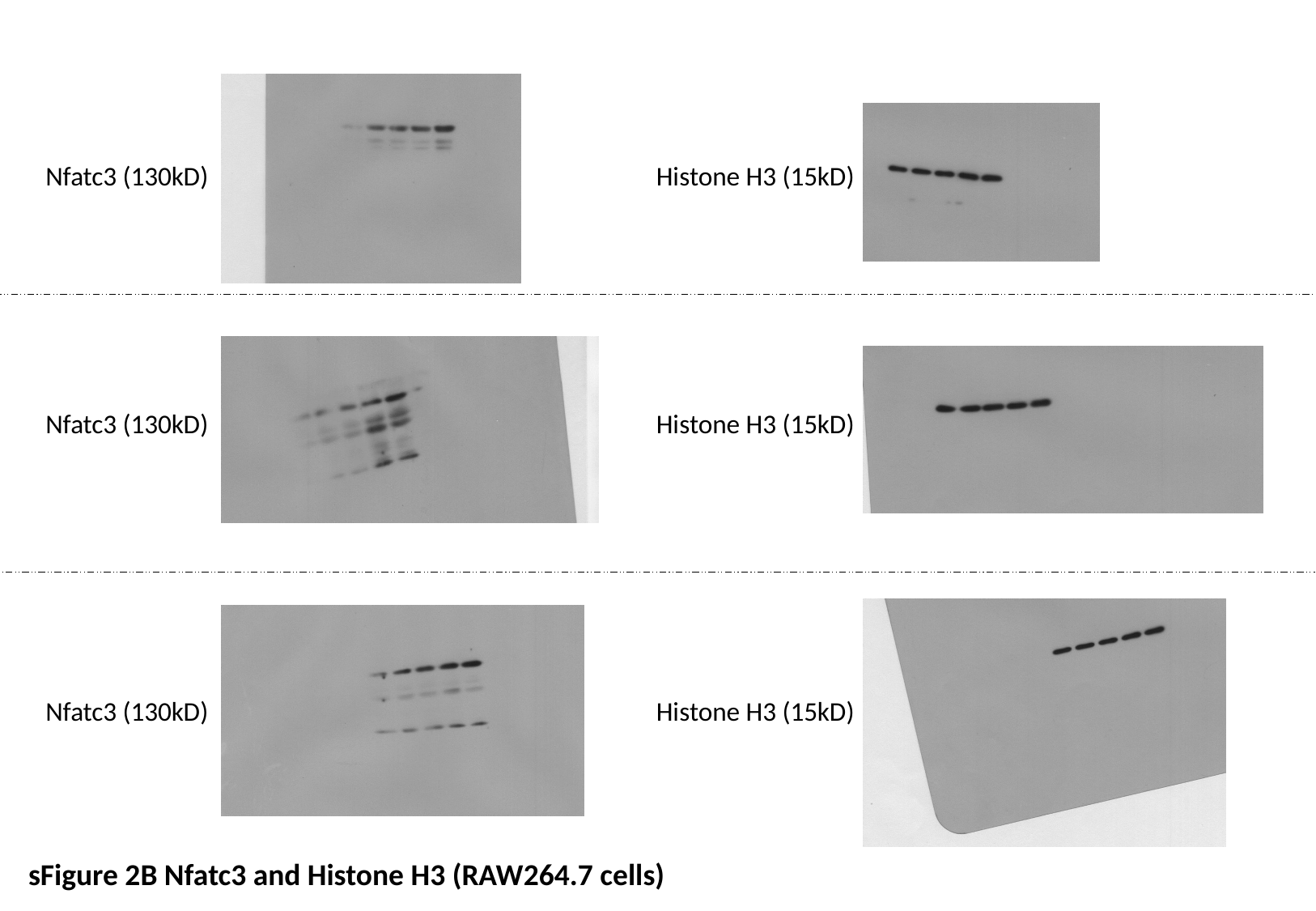

Histone H3 (15kD)
Nfatc3 (130kD)
Histone H3 (15kD)
Nfatc3 (130kD)
Histone H3 (15kD)
Nfatc3 (130kD)
sFigure 2B Nfatc3 and Histone H3 (RAW264.7 cells)

## Slide 19
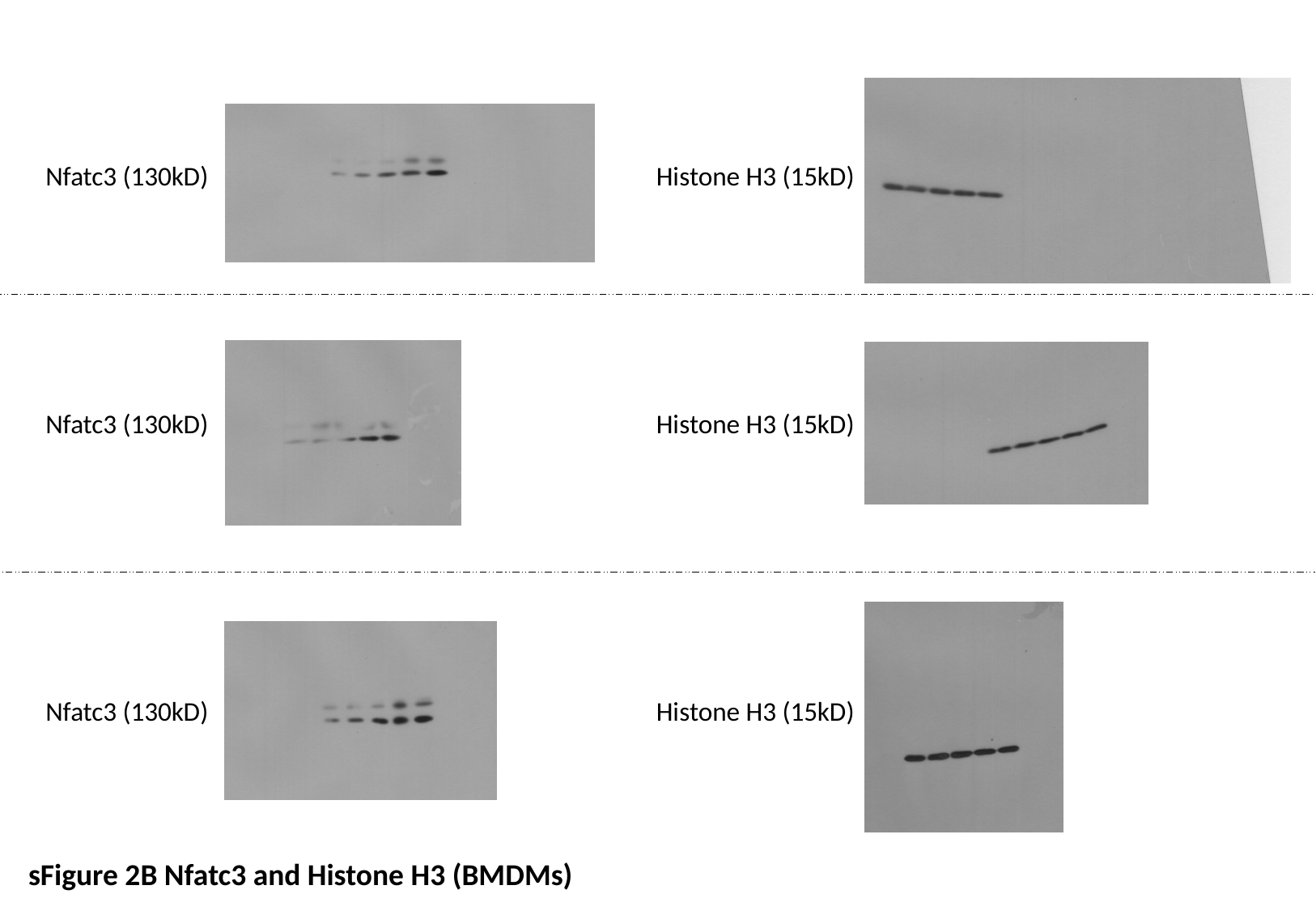

Histone H3 (15kD)
Nfatc3 (130kD)
Histone H3 (15kD)
Nfatc3 (130kD)
Histone H3 (15kD)
Nfatc3 (130kD)
sFigure 2B Nfatc3 and Histone H3 (BMDMs)

## Slide 20
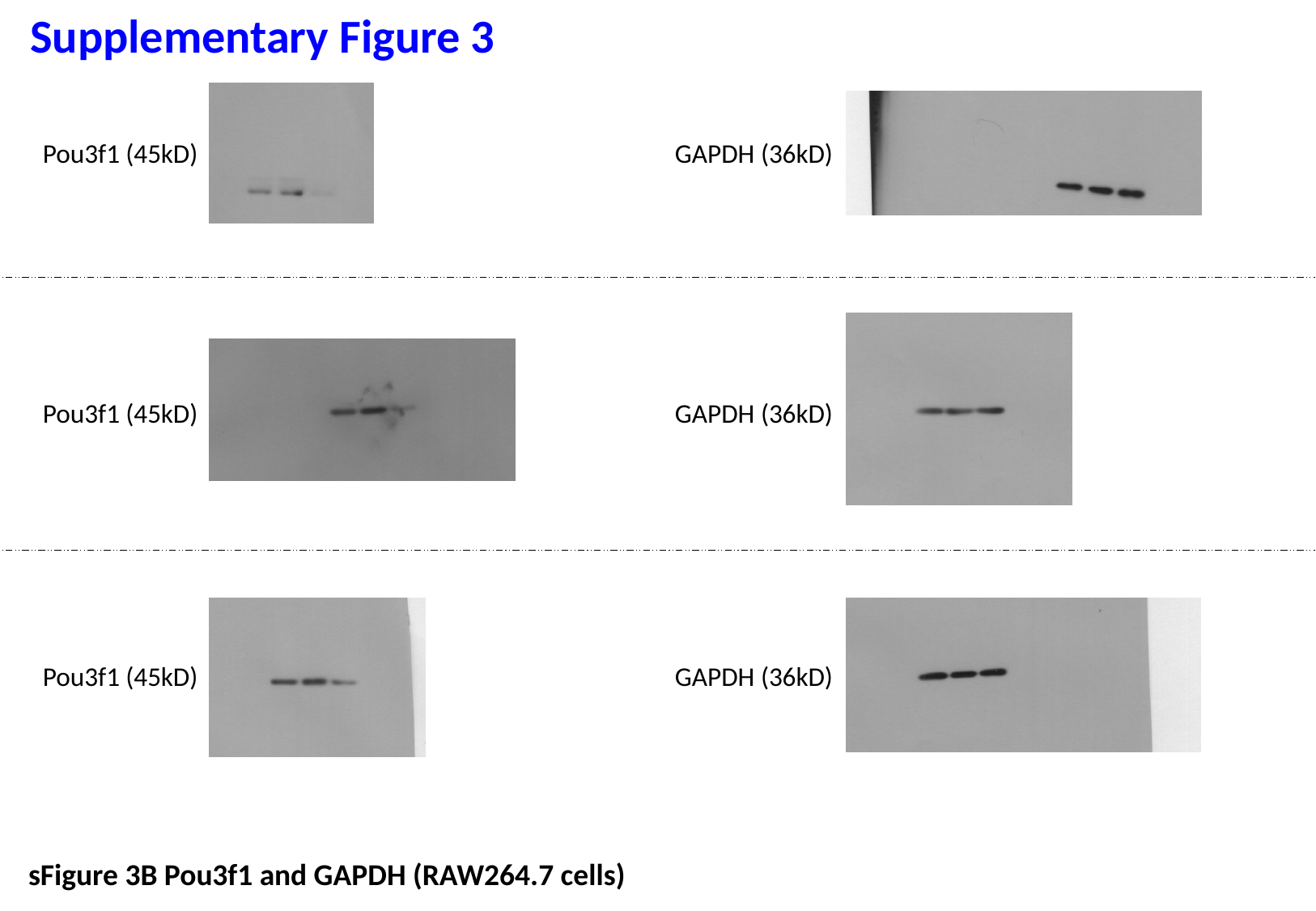

Supplementary Figure 3
Pou3f1 (45kD)
GAPDH (36kD)
Pou3f1 (45kD)
GAPDH (36kD)
Pou3f1 (45kD)
GAPDH (36kD)
sFigure 3B Pou3f1 and GAPDH (RAW264.7 cells)
